# Supplementary material for: Neutrophil–lymphocyte ratio across psychiatric diagnoses: a cross-sectional study using electronic health records
Source: BMJ Open. 2020 Jul 19;10(7):e036859. doi: 10.1136/bmjopen-2020-036859 (PMC7371128; doi:10.1136/bmjopen-2020-036859)
Supplement: Supplementary data [file bmjopen-2020-036859supp001.pdf]

Supplementary Materials

Table S1: NLR between controls and ICD-10 diagnoses using a log-linked gamma generalised linear model.

	Estimate	Std. Error	z value	Pr(>|z|)	
(Intercept)	0.5243	0.023	22.55	< 2e-16	***
F00	0.1483	0.031	4.834	1.34E-06	***
F01	0.2366	0.081	2.922	0.00348	**
F02	0.2687	0.101	2.657	0.00789	**
F03	0.1166	0.064	1.832	0.06699	.
F04	-0.455	0.283	-1.61	0.10796	
F05	0.2782	0.094	2.964	0.00303	**
F06	0.1643	0.048	3.431	0.0006	***
F07	0.1374	0.101	1.362	0.17319	
F09	0.0506	0.108	0.47	0.63829	
F10	0.158	0.021	7.649	2.03E-14	***
F11	0.0185	0.037	0.504	0.614	
F12	0.043	0.05	0.865	0.38709	
F13	0.0085	0.11	0.077	0.93867	
F14	-0.098	0.091	-1.08	0.28035	
F15	-0.159	0.142	-1.12	0.26347	
F16	-0.039	0.2	-0.2	0.84467	
F17	-0.314	0.49	-0.64	0.52183	
F18	-0.112	0.347	-0.32	0.74688	
F19	0.0642	0.036	1.785	0.07423	.
F20	0.0944	0.018	5.331	9.74E-08	***
F21	-0.168	0.186	-0.9	0.36673	
F22	0.0539	0.041	1.308	0.19077	
F23	0.1595	0.029	5.505	3.69E-08	***
F25	0.0737	0.024	3.067	0.00216	**
F28	0.1657	0.076	2.171	0.02992	*
F29	0.1263	0.024	5.207	1.92E-07	***
F30	0.2002	0.043	4.67	3.01E-06	***
F31	0.1161	0.023	5.123	3.01E-07	***
F32	0.1318	0.02	6.514	7.31E-11	***
F33	0.1439	0.029	4.938	7.89E-07	***
F34	-0.113	0.103	-1.1	0.27259	
F38	0.4065	0.127	3.196	0.00139	**
F39	-0.014	0.116	-0.12	0.90559	
F40	-0.137	0.092	-1.49	0.13529	
F41	0.0666	0.031	2.135	0.03277	*
F42	-0.017	0.033	-0.51	0.60838	
F43	0.0829	0.029	2.825	0.00473	**
F44	0.0348	0.049	0.716	0.47408	

F45	0.0248	0.063	0.397	0.69152	
F48	-0.047	0.035	-1.36	0.17526	
F50	0.0274	0.016	1.747	0.08069	.
F52	-0.206	0.2	-1.03	0.30387	
F53	0.0962	0.056	1.728	0.084	.
F55	-0.093	0.49	-0.19	0.84988	
F60	0.0883	0.025	3.476	0.00051	***
F61	0.1759	0.083	2.127	0.03343	*
F64	-0.105	0.283	-0.37	0.7107	
F68	0.1445	0.347	0.417	0.67677	
F69	0.0032	0.186	0.017	0.98645	
F70	0.1351	0.058	2.344	0.01909	*
F71	0.097	0.113	0.855	0.39248	
F72	0.101	0.245	0.412	0.68036	
F78	0.192	0.49	0.392	0.69525	
F79	0.3979	0.201	1.984	0.04731	*
Age	0.005	2E-04	20.22	< 2e-16	***
Black ethnicity	-0.226	0.019	-11.9	< 2e-16	***
Mixed/other ethnicity	-0.022	0.021	-1.07	0.28411	
White ethnicity	0.0921	0.018	4.998	5.79E-07	***
Gender Male	-0.01	0.008	-1.31	0.19134	
Gender Not Specified	-0.425	0.347	-1.22	0.22082	
Gender Other	-0.096	0.49	-0.2	0.84531	
Antipsychotics	0.0375	0.013	2.963	0.00305	**
Antidepressants	0.0115	0.009	1.247	0.21246	
Mood stabilisers	-0.004	0.016	-0.23	0.81925	
Hypnotics	0.025	0.01	2.54	0.01107	*

Signif. codes: ‘***’ 0.001 ‘**’ 0.01 ‘*’ 0.05 ‘.’ 0.1

Table S2: NLR between controls and ICD-10 diagnoses using a log-linked gamma generalised linear model uncorrected for covariates.

	Estimate	Std. Error	z value	Pr(>|z|)	
(Intercept)	0.73521	0.00818	89.878	< 2e-16	***
F00	0.39918	0.03013	13.25	< 2e-16	***
F01	0.50716	0.08349	6.075	1.24E-09	***
F02	0.45036	0.10486	4.295	1.75E-05	***
F03	0.39547	0.06504	6.08	1.20E-09	***
F04	-0.42323	0.29581	-1.431	0.152494	
F05	0.49682	0.09713	5.115	3.14E-07	***
F06	0.22881	0.04887	4.682	2.84E-06	***
F07	0.26206	0.10486	2.499	0.012453	*

F09	0.07505	0.11206	0.67	0.50304	
F10	0.26071	0.01911	13.64	< 2e-16	***
F11	0.10116	0.03687	2.744	0.006068	**
F12	-0.03256	0.05018	-0.649	0.516471	
F13	0.08988	0.11481	0.783	0.433742	
F14	-0.07706	0.09386	-0.821	0.411629	
F15	-0.08964	0.14807	-0.605	0.544945	
F16	-0.05719	0.20925	-0.273	0.784626	
F17	-0.1782	0.51222	-0.348	0.727919	
F18	-0.20156	0.36224	-0.556	0.577916	
F19	0.08136	0.03628	2.243	0.024911	*
F20	0.09383	0.01256	7.47	8.04E-14	***
F21	-0.10163	0.19375	-0.525	0.599898	
F22	0.14609	0.04094	3.568	0.000359	***
F23	0.09937	0.02676	3.713	0.000205	***
F25	0.06628	0.02038	3.252	0.001144	**
F28	0.14642	0.07853	1.864	0.062257	.
F29	0.06177	0.02095	2.948	0.003196	**
F30	0.19001	0.04234	4.488	7.20E-06	***
F31	0.16235	0.01815	8.944	< 2e-16	***
F32	0.19281	0.01779	10.838	< 2e-16	***
F33	0.26541	0.02785	9.528	< 2e-16	***
F34	-0.05149	0.10711	-0.481	0.630725	
F38	0.55561	0.13249	4.194	2.75E-05	***
F39	0.03868	0.12099	0.32	0.749236	
F40	-0.10782	0.09546	-1.13	0.258666	
F41	0.1576	0.03059	5.153	2.57E-07	***
F42	0.06606	0.03239	2.039	0.041423	*
F43	0.0728	0.02876	2.531	0.011369	*
F44	0.10234	0.04996	2.049	0.040501	*
F45	0.08563	0.06454	1.327	0.184609	
F48	0.01074	0.03595	0.299	0.765057	
F50	0.03339	0.01392	2.399	0.016452	*
F52	-0.17707	0.20925	-0.846	0.397441	
F53	0.09034	0.05648	1.6	0.109689	
F55	0.05383	0.51222	0.105	0.9163	
F60	0.15522	0.02323	6.682	2.35E-11	***
F61	0.20589	0.08575	2.401	0.01635	*
F64	-0.12422	0.29581	-0.42	0.674545	
F68	0.17984	0.36224	0.496	0.619568	
F69	0.02839	0.19375	0.147	0.883506	
F70	0.15574	0.05894	2.642	0.008231	**
F71	0.09172	0.11778	0.779	0.436162	
F72	0.18543	0.25621	0.724	0.469227	
F78	0.27214	0.51222	0.531	0.595216	
F79	0.53105	0.20925	2.538	0.011152	*

Signif. codes: '***' 0.001 '**' 0.01 '*' 0.05 '.' 0.1

Table S3: NLR between ICD-10 diagnoses with elevated NLR in first model using a log-linked gamma generalised linear model.

	Estimate	Std. Error	z value	Pr(>|z|)	
(Intercept)	0.6081	0.0453	13.412	< 2e-16	***
F01	0.0927	0.0872	1.064	0.28742	
F02	0.1411	0.1075	1.312	0.18937	
F05	0.1351	0.1001	1.351	0.17685	
F06	0.0472	0.0567	0.834	0.40448	
F10	0.0371	0.0359	1.033	0.30155	
F20	-0.0104	0.0337	-0.309	0.75758	
F23	0.0608	0.0423	1.44	0.14991	
F25	-0.0333	0.0376	-0.887	0.37531	
F28	0.0617	0.0841	0.734	0.46269	
F29	0.0288	0.0391	0.736	0.46202	
F30	0.0979	0.0532	1.839	0.06587	.
F31	0.0027	0.0366	0.074	0.9411	
F32	0.0115	0.0357	0.323	0.74672	
F33	0.0154	0.0407	0.378	0.7053	
F38	0.2727	0.1344	2.029	0.0425	*
F41	-0.0555	0.0435	-1.278	0.20121	
F43	-0.027	0.0427	-0.632	0.52728	
F60	-0.0299	0.0395	-0.757	0.44903	
F70	0.0292	0.0662	0.441	0.65913	
F79	0.2722	0.2093	1.301	0.19336	
Age	0.0059	0.0004	16.212	< 2e-16	***
Black ethnicity	-0.2412	0.022	-10.945	< 2e-16	***
Mixed/other ethnicity	-0.0005	0.0262	-0.019	0.98449	
White ethnicity	0.1052	0.0215	4.897	9.73E-07	***
Gender Male	-0.0322	0.0105	-3.069	0.00215	**
Gender Not Specified	-0.1816	0.5072	-0.358	0.72033	
Antipsychotics	0.0331	0.0161	2.055	0.03992	*
Antidepressants	0.0134	0.0113	1.187	0.23507	
Mood stabilisers	-0.0079	0.0168	-0.472	0.63677	
Hypnotics	0.0299	0.0113	2.635	0.00842	**

Signif. codes: '***' 0.001 '**' 0.01 '*' 0.05 '.' 0.1

Table S4: NLR between ICD-10 diagnoses with elevated NLR in first model using a log-linked gamma generalised linear model uncorrected for covariates.

	Estimate	Std. Error	z value	Pr(>|z|)	
(Intercept)	1.1344	0.03027	37.471	< 2e-16	***
Short_DiagF01	0.10798	0.09188	1.175	0.239912	
Short_DiagF02	0.05117	0.11327	0.452	0.651437	
Short_DiagF05	0.09764	0.10549	0.926	0.354694	
Short_DiagF06	-0.17037	0.05871	-2.902	0.00371	**
Short_DiagF10	-0.13847	0.03524	-3.929	8.52E-05	***
Short_DiagF20	-0.30535	0.03187	-9.582	< 2e-16	***
Short_DiagF23	-0.29982	0.0403	-7.439	1.01E-13	***
Short_DiagF25	-0.3329	0.036	-9.246	< 2e-16	***
Short_DiagF28	-0.25276	0.08699	-2.906	0.003663	**
Short_DiagF29	-0.33741	0.03636	-9.28	< 2e-16	***
Short_DiagF30	-0.20917	0.05289	-3.955	7.67E-05	***
Short_DiagF31	-0.23684	0.03468	-6.829	8.55E-12	***
Short_DiagF32	-0.20638	0.03448	-5.986	2.15E-09	***
Short_DiagF33	-0.13377	0.0411	-3.255	0.001135	**
Short_DiagF38	0.15643	0.14135	1.107	0.268444	
Short_DiagF41	-0.24158	0.04317	-5.596	2.19E-08	***
Short_DiagF43	-0.32638	0.04178	-7.812	5.61E-15	***
Short_DiagF60	-0.24396	0.03784	-6.448	1.14E-10	***
Short_DiagF70	-0.24345	0.06805	-3.578	0.000347	***
Short_DiagF79	0.13187	0.2204	0.598	0.549634	

Signif. codes: '***' 0.001 '**' 0.01 '*' 0.05 '.' 0.1

Table S5: Association between NLR and mortality using a log-linked gamma generalised linear model.

	Estimate	Std. Error	z value	Pr(>|z|)	
(Intercept)	0.629	0.042	15.05	< 2e-16	***
Mortality	0.103	0.019	5.547	2.90E-08	***
F01	0.075	0.086	0.881	0.378368	
F02	0.112	0.106	1.06	0.289297	
F03	-0.03	0.069	-0.44	0.662089	
F04	-0.55	0.288	-1.92	0.054981	.
F05	0.123	0.098	1.251	0.211037	
F06	0.048	0.055	0.867	0.385872	
F07	0.01	0.106	0.092	0.9267	
F09	-0.06	0.113	-0.5	0.617792	
F10	0.04	0.035	1.139	0.254815	

F11	-0.1	0.046	-2.19	0.028768	*
F12	-0.05	0.058	-0.93	0.352593	
F13	-0.09	0.115	-0.79	0.427049	
F14	-0.2	0.096	-2.06	0.039388	*
F15	-0.26	0.147	-1.75	0.080089	.
F16	-0.14	0.205	-0.68	0.499768	
F17	-0.43	0.498	-0.86	0.391132	
F18	-0.21	0.353	-0.6	0.5495	
F19	-0.04	0.046	-0.84	0.40397	
F20	-0.01	0.032	-0.3	0.760888	
F21	-0.27	0.19	-1.41	0.159867	
F22	-0.06	0.049	-1.27	0.20328	
F23	0.061	0.041	1.495	0.134955	
F25	-0.03	0.036	-0.86	0.392265	
F28	0.061	0.082	0.747	0.45498	
F29	0.027	0.037	0.709	0.478204	
F30	0.098	0.052	1.895	0.058073	.
F31	0.007	0.035	0.197	0.844041	
F32	0.021	0.035	0.608	0.543242	
F33	0.025	0.04	0.623	0.533192	
F34	-0.22	0.108	-2.07	0.038898	*
F38	0.29	0.132	2.199	0.027893	*
F39	-0.12	0.121	-0.98	0.327305	
F40	-0.24	0.098	-2.47	0.013716	*
F41	-0.04	0.042	-1.04	0.300219	
F42	-0.12	0.044	-2.73	0.006259	**
F43	-0.02	0.041	-0.51	0.611605	
F44	-0.07	0.057	-1.31	0.191077	
F45	-0.09	0.07	-1.25	0.211284	
F48	-0.16	0.046	-3.37	0.000762	***
F50	-0.08	0.035	-2.42	0.015461	*
F52	-0.3	0.205	-1.47	0.141943	
F53	-0.01	0.063	-0.22	0.826974	
F55	-0.19	0.498	-0.39	0.697018	
F60	-0.02	0.038	-0.57	0.571013	
F61	0.076	0.088	0.862	0.388455	
F64	-0.21	0.289	-0.72	0.472579	
F68	0.052	0.352	0.149	0.881745	
F69	-0.1	0.19	-0.53	0.598212	
F70	0.036	0.065	0.559	0.576053	
F71	9E-04	0.118	0.008	0.993735	
F72	0.001	0.25	0.005	0.996367	
F78	0.102	0.498	0.205	0.837852	
F79	0.289	0.205	1.411	0.158226	
Age	0.005	3E-04	16.29	< 2e-16	***
Black ethnicity	-0.24	0.02	-12.1	< 2e-16	***

Mixed/other ethnicity	-0	0.023	-0.16	0.87651	
White ethnicity	0.094	0.019	4.975	6.53E-07	***
Gender Male	-0.03	0.009	-3.14	0.00168	**
Gender Not Specified	-0.42	0.352	-1.2	0.229188	
Gender Other	-0.09	0.497	-0.19	0.853287	
Antipsychotics	0.037	0.013	2.887	0.003889	**
Antidepressants	0.008	0.009	0.88	0.378798	
Mood stabilisers	-0.01	0.016	-0.32	0.748271	
Hypnotics	0.025	0.01	2.479	0.013183	*

Signif. codes: ‘***’ 0.001 ‘**’ 0.01 ‘*’ 0.05 ‘.’ 0.1
