# Supplementary material for: Neutrophil–lymphocyte ratio across psychiatric diagnoses: a cross-sectional study using electronic health records
Source: BMJ Open. 2020 Jul 19;10(7):e036859. doi: 10.1136/bmjopen-2020-036859 (PMC7371128; doi:10.1136/bmjopen-2020-036859)
Supplement: Author's manuscript [file bmjopen-2020-036859.draft_revisions.pdf]

BMJ Open

BMJ Open is committed to open peer review. As part of this commitment we make the peer review history of every article we publish publicly available.

When an article is published we post the peer reviewers' comments and the authors' responses online. We also post the versions of the paper that were used during peer review. These are the versions that the peer review comments apply to.

The versions of the paper that follow are the versions that were submitted during the peer review process. They are not the versions of record or the final published versions. They should not be cited or distributed as the published version of this manuscript.

BMJ Open is an open access journal and the full, final, typeset and author-corrected version of record of the manuscript is available on our site with no access controls, subscription charges or pay-per-view fees (http://bmjopen.bmj.com).

If you have any questions on BMJ Open's open peer review process please email info.bmjopen@bmj.com

BMJ Open

Neutrophil-lymphocyte ratio across psychiatric diagnoses: An electronic health record investigation

Journal:	*BMJ Open*
Manuscript ID	bmjopen-2020-036859
Article Type:	Original research
Date Submitted by the Author:	10-Jan-2020
Complete List of Authors:	Brinn, Aimee; King's College London<br>Stone, James; King's College London,
Keywords:	MENTAL HEALTH, Schizophrenia & psychotic disorders < PSYCHIATRY, Depression & mood disorders < PSYCHIATRY, Personality disorders < PSYCHIATRY, Anxiety disorders < PSYCHIATRY

SCHOLARONE™
Manuscripts

I, the Submitting Author has the right to grant and does grant on behalf of all authors of the Work (as defined in the below author licence), an exclusive licence and/or a non-exclusive licence for contributions from authors who are: i) UK Crown employees; ii) where BMJ has agreed a CC-BY licence shall apply, and/or iii) in accordance with the terms applicable for US Federal Government officers or employees acting as part of their official duties; on a worldwide, perpetual, irrevocable, royalty-free basis to BMJ Publishing Group Ltd ("BMJ") its licensees and where the relevant Journal is co-owned by BMJ to the co-owners of the Journal, to publish the Work in this journal and any other BMJ products and to exploit all rights, as set out in our licence.

The Submitting Author accepts and understands that any supply made under these terms is made by BMJ to the Submitting Author unless you are acting as an employee on behalf of your employer or a postgraduate student of an affiliated institution which is paying any applicable article publishing charge ("APC") for Open Access articles. Where the Submitting Author wishes to make the Work available on an Open Access basis (and intends to pay the relevant APC), the terms of reuse of such Open Access shall be governed by a Creative Commons licence – details of these licences and which Creative Commons licence will apply to this Work are set out in our licence referred to above.

Other than as permitted in any relevant BMJ Author's Self Archiving Policies, I confirm this Work has not been accepted for publication elsewhere, is not being considered for publication elsewhere and does not duplicate material already published. I confirm all authors consent to publication of this Work and authorise the granting of this licence.

For peer review only

Neutrophil-lymphocyte ratio across psychiatric diagnoses: An electronic health record investigation

Aimee Brinn[1], James M Stone MBBS PhD FRCPsych[1] *

1) King's College London Institute of Psychiatry, Psychology and Neuroscience, De Crespigny Park, London, SE5 8AF

* Address correspondence to: Dr James Stone, Centre for Neuroimaging Sciences, Institute of Psychiatry Psychology & Neuroscience, 16 De Crespigny Park, London SE5 8AF, UK. Email: james.m.stone@kcl.ac.uk.

Keywords: NLR, neutrophil lymphocyte ratio, inflammation, psychiatric diagnoses, mental illness

Abstract word count: 240

Main body word count: 3387

For peer review only

Abstract

Objectives: The main objective of this study was to compare neutrophil-lymphocyte ratio, a marker of systemic inflammation, between patients diagnosed with ICD-10 psychiatric disorders and control participants.

Design: A cross-sectional methodology was employed to retrospectively analyse electronic health records and records derived from a national health survey.

Setting: A secondary mental health care service consisting of four boroughs in South London.

Participants: A diverse sample of 13,888 psychiatric patients extracted from South London and Maudsley electronic health records database and 3,920 control participants extracted from National Health and Nutrition Survey (2015-2016) were included in the study.

Primary and secondary outcome measures: Primary: NLR levels in patients with mental health diagnoses, NLR between patients with different mental health diagnoses. Secondary: Relationship of NLR to length of hospitalisation and to mortality.

Results: NLR was elevated compared to controls in patients with diagnoses including dementia, alcohol dependence, schizophrenia, bipolar affective disorder, depression, non-phobic anxiety disorders, and mild mental retardation ($p < 0.05$). NLR also correlated with age, antipsychotic use and hypnotic use. NLR was found to be higher in individuals of “White” ethnicity and lower in individuals of “Black” ethnicity. Elevated NLR was associated with increased mortality ($\beta = 0.103$, $p = 2.9e\text{-}08$) but not with hospital admissions or face-to-face contacts.

Conclusions: Elevated NLR may reflect a transdiagnostic pathological process occurring in a subpopulation of psychiatric patients. NLR may be useful to identify and stratify patients who could benefit from adjunctive anti-inflammatory treatment.

For peer review only - http://bmjopen.bmj.com/site/about/guidelines.xhtml

Article Summary

Strengths and limitations of this study

- Largest study to date of cross-diagnostic neutrophil-lymphocyte measurements in a psychiatric population.
- Sample is representative of diverse adult psychiatric patients in South London.
- Systematic differences between patients and controls reduce the validity of these comparisons.
- This study was retrospective and thus, confounding measures such as body mass index, smoking status and diet were unavailable.

Conflict of Interest

The authors report no conflict of interest.

Financial Support

This paper represents independent research funded by the National Institute for Health Research (NIHR) Biomedical Research Centre at South London and Maudsley NHS Foundation Trust and King's College London. The views expressed are those of the authors and not necessarily those of the NHS, the NIHR or the Department of Health and Social Care.

Data Sharing Statement

Because of their clinical source and under the terms of Ethics and Information Governance approvals, datasets derived from CRIS have to remain within the SLaM firewall. However, all data used for this study will be made accessible on request to cris.administrator@slam.nhs.uk subject to setting up an appropriate SLaM honorary contract or research passport.

For peer review only

For peer review only - http://bmjopen.bmj.com/site/about/guidelines.xhtml

Introduction

Inflammation is a key component of our immune response to physiological injury or infection (Xiao, 2017). Low-grade systemic inflammation is an attenuated, but persistent, form of the inflammatory response and has been found to be prevalent across a range of psychiatric diagnoses, including psychotic, mood, neurotic, and personality, disorders (Osimo et al, 2018).

Blood biomarkers are the most commonly used method to study inflammatory processes in psychiatry (Fond et al, 2018, Pinto et al, 2017), but many of these are costly or difficult to collect routinely. The neutrophil to lymphocyte ratio (NLR) can be calculated from a full blood count and is thus cheaper and more readily available than cytokine testing (Gibson et al, 2007). NLR has been suggested to be a marker of the intensity of physiological stress present in acutely ill patients, and a measure of systemic inflammation (Zahorec, 2001). As it constitutes parameters from both innate (neutrophil) and adaptive (lymphocyte) immune systems, it may be less affected by confounding variables, such as exercise, compared to other commonly used inflammatory biomarkers (Gibson et al, 2007). NLR has demonstrated reliable prognostic value across a range of physical health disorders (Afari & Bhat, 2016, Chandrashekara et al, 2017, Koh et al, 2015, Wang et al, 2017), highlighting a positive association between systemic inflammation and worse clinical outcomes.

The most studied inflammatory biomarkers in psychiatry, interleukin-6 (IL-6), tumour necrosis factor (TNF) and c-reactive protein (CRP), are elevated in patients with schizophrenia, bipolar disorder and depression, compared to controls (Pinto et al, 2017).

For peer review only - http://bmjopen.bmj.com/site/about/guidelines.xhtml

NLR also appears to be elevated in major psychiatric disorders compared to controls (Karageorgiou et al, 2018 ,Mazza et al, 2018, Mazza et al, 2019a). However, the vast proportion of studies have studied a single disorder in comparison to healthy controls, or at best, two different psychiatric disorders (Pinto et al, 2017). True transdiagnostic studies are rare with only three studies to date comparing NLR between psychiatric diagnoses (Baykan et al, 2018, Mazza et al, 2019b, Ozdin et al, 2017).

Of the NLR studies comparing different psychiatric diagnoses, Ozdin et al (2017) reported that patients with schizophrenia, experiencing a psychotic episode, had higher NLR than patients with manic-phase bipolar disorder (BD), suggesting there are detectable differences in NLR across diagnosis and symptomatology. Mazza et al (2019b) found patients with manic-phase BD had higher NLR than patients with major depressive disorder (MDD). They also found significant differences between manic-phase and depressive-phase BD but not between depressive-phase BD and MDD suggesting that NLR may be elevated in mania but not depression. Lastly, Baykan et al (2018) found NLR to be significantly elevated in elderly patients with Alzheimer's (AD), compared to elderly patients with MDD. Shared symptomatology between AD and MDD is problematic for clinicians differentiating between the disorders (Baykan et al, 2018) and the authors suggested that NLR may be an effective diagnostic marker to aid discrimination.

The current evidence base is limited and inconsistent. Further research is required to investigate the role of inflammatory processes in psychiatric illness. Differentially elevated inflammation between different diagnoses may suggest inflammatory processes are more common in specific disorders. Alternatively, comparable elevation between diagnoses may

For peer review only - http://bmjopen.bmj.com/site/about/guidelines.xhtml

suggest common aetiological pathways, such as shared immunological genetic vulnerabilities and inflammatory processes. Patients with elevated NLR may represent a subtype of psychiatric illness that does not fit within current diagnostic categories. Lastly, systemic inflammation may be a by-product of mental health difficulties, reflecting a non-specific comorbid physiological stress.

In this study, we aimed to characterise NLR across a range of ICD-10 (World Health Organisation, 1992) psychiatric diagnoses and to investigate the relationship between NLR and adverse clinical outcomes.

Methods

Design

This study employed a cross-sectional methodology to analyse electronic health records extracted using the Clinical Record Interactive Search (CRIS) tool (Stewart et al, 2009). We retrospectively analysed neutrophil and lymphocyte counts of psychiatric patients using whole blood count data. For comparison, a control group were obtained from the National Health and Nutrition Examination Survey (Centers for Disease Control and Prevention: National Center for Health Statistics, 2016).

Clinical Record Interactive Search

The Clinical Record Interactive Search (CRIS) application was developed in 2007, with funding from the British National Institute for Health Research. The application generates a data resource consisting of anonymised electronic health records of service users of the SLaM NHS Foundation Trust (Stewart et al, 2009). These records are contained in the

For peer review only - http://bmjopen.bmj.com/site/about/guidelines.xhtml

Biomedical Research Centre (BRC) case register, which is updated every 24 hours. CRIS was used to access patient data, using SQL management studio as a platform for data extraction.

CRIS has ethical approval for usage as an anonymised database for secondary analysis (reference 18/SC/0372). This project was approved by the CRIS Oversight Committee.

Patient Sample

The SLaM BRC case register includes all patients who have accessed secondary mental health care services within a catchment area of four south London boroughs (Lambeth, Southwark, Lewisham and Croydon). SLaM is one of the largest mental healthcare organisations in Europe, providing care to 1.3 million people (Lopez-Morinigo et al, 2018).

We extracted data from records for all patients who accessed SLaM secondary mental health care between 01-01-2007, when CRIS was established, and 20-11-2018, when our data were extracted.

Data were extracted for all patients meeting the following inclusion criteria:

- ≥ 16 years old at earliest recorded blood test
- ICD-10 Primary Diagnosis F01-F79
- Recorded neutrophil count
- Recorded lymphocyte count

13,888 patients met our inclusion criteria and were included in the sample.

For peer review only

Control Sample

The control group comprised a sample of participants from the 2015–2016 National Health and Nutrition Survey (Centers for Disease Control and Prevention: National Center for Health Statistics, 2016). The original sample included 9,971 participants from across 30 locations in the United States. Hispanic, Black and Asian individuals are overrepresented in the sample, as are individuals at or below 185 percent of the Department of Health and Human Services poverty guidelines (Burwell, 2015) and those aged 80 and over.

We included individuals meeting the following inclusion criteria:

- ≥16 years old at time of NHANES (2015-2016) survey
- Recorded neutrophil count
- Recorded lymphocyte count

Office for National Statistics (ONS) (Office for National Statistics, 2009) guidelines do not code 'Hispanic' as a distinct ethnic group. 'Hispanic' was therefore not a distinct ethnic group in the CRIS database. For ease of sample comparison, we excluded Hispanic participants from our study. 3,920 participants met our criteria and were included in the control sample.

Variables

Demographics

Demographic variables were retrieved from structured fields in the CRIS database and included age at earliest blood test, gender and ethnicity. Ethnicity was coded using ONS

For peer review only - http://bmjopen.bmj.com/site/about/guidelines.xhtml

guidelines (Office for National Statistics, 2009). To facilitate comparison with the NHANES sample, we combined 'Other' and 'Mixed' ethnicity codes into one category 'Other/Mixed'.

Neutrophil to lymphocyte ratio

Absolute neutrophil and lymphocyte counts were obtained from routine blood testing data, attained at the point of patient entry to SLaM secondary care services. NLR was calculated for each patient by dividing neutrophil count by lymphocyte count. We extracted earliest available blood count values to minimise potential confounding effects of secondary healthcare treatment.

ICD-10 diagnosis

We included diagnoses from eight ICD-10 diagnostic blocks in this study: Organic, including symptomatic, mental disorders (F01-F09), Mental and behavioural disorders due to psychoactive substance use (F10-F19), Schizophrenia, schizotypal and delusional disorders (F20-F29), mood [affective] disorders (F30-F39), Neurotic, stress-related and somatoform disorders (F40-F49), Behavioural syndromes associated with physiological disturbances and physical factors (F50-59), Disorders of adult personality and behaviour (F60-F69) and Mental retardation (F70-F79). Diagnostic variables were defined by the primary diagnosis assigned to the patient closest to their blood test result.

We excluded patients with diagnoses in the Disorders of psychological development (F80-F89) and Behavioural/emotional disorders with onset usually occurring in childhood and adolescence (F90-F98) diagnostic blocks as most patients were under 16 years old. We also

For peer review only - http://bmjopen.bmj.com/site/about/guidelines.xhtml

excluded patients in the Unspecified mental disorder (F99) block due to ambiguity of the diagnosis.

Adverse clinical outcomes

We defined 'number of overnight bed stays' as the total number of overnight hospital stays for each patient since the date of the blood test. We defined 'number of face-to-face events' as the number of clinical face-to-face contacts, such as hospital appointments, for each patient since the date of the blood test. Both measures were adjusted for patient time in services since the date of the blood test. Death was recorded as a dichotomous mortality variable.

Medication

The medication prescribed to the patient within 1 year of the blood test date was extracted. Medications were categorised into four classes: antipsychotics, antidepressants, mood stabilisers and hypnotics.

Statistical Analysis

We conducted all statistical analyses for this study using R (version 3.6). We analysed data distribution by use of histogram. We report the mean and standard deviation (SD) for normally distributed data.

Because NLR data demonstrated an exponential distribution (see Figures 1 and 2),we conducted regression analyses using a log-linked gamma-family generalised linear model. For each analysis, the shape parameter of the model was estimated using the gamma.shape

For peer review only - http://bmjopen.bmj.com/site/about/guidelines.xhtml

function from the MASS package, and this was used to set the dispersion parameter as 1/(maximum likelihood estimate). We ran the following models: 1) Control NLR as dependent variable vs. NLR in ICD-10 diagnoses; 2) NLR between all ICD-10 diagnoses that were significantly different from control NLR from Model 1. In Models 1 and 2 age, ethnicity, gender and medication were included as independent variables. We then ran three separate models within the CRIS dataset only to investigate the association of NLR with clinical outcome measures. These models set NLR as the dependent variable vs death, hospital admissions or numbers of face-to face contact. ICD-10 diagnosis, age, ethnicity, gender and medication were included as independent variables.

Patient and Public Involvement

No patient involved.

Results

Demographic Results

The mean ages of patients and controls were 40.1 (SD: 16.57) and 46.9 (SD: 19.47) respectively. Distribution of demographic variables are displayed in Table 1.

There was no relationship between date of blood test (from which the NLR value was derived) and NLR value across the patient sample (β = -4.76e-06, p = 0.692) suggesting that NLR measurement remained stable across the defined time period (2007-2018).

Model 1: NLR between controls and patients

For peer review only - http://bmjopen.bmj.com/site/about/guidelines.xhtml

NLR in the following ICD-10 diagnoses were significantly higher than controls: F00, Alzheimer's disease (β = 0.15, p = 1.3e-06); F01, vascular dementia (β = 0.24, p = 0.003); F02 dementia in other diseases (β = 0.27, p = 0.008); F05, delirium (β =0.28, p = 0.003); F06, other mental disorders due to brain damage and dysfunction (β = 0.16, p = 0.0006); F10, mental and behavioural disorders due to alcohol (β = 0.16, p = 2.0e-14); F20, schizophrenia (β = 0.09, p = 9.7e-08); F23, acute and transient psychotic disorders (β = 0.16, p = 3.7e-08); F25, schizoaffective disorders (β = 0.07, p = 0.002); F28, other nonorganic psychotic disorders (β = 0.17, p = 0.03); F29, unspecified nonorganic psychotic disorders (β = 0.13, p = 1.9e-07); F30, manic episode (β = 0.20, p = 3.0e-06); F31, bipolar affective disorder (β = 0.12, p = 3.0e-07); F32, depressive episode (β = 0.13, p =7.31e-11); F33, recurrent depressive disorder (0.14, p = 7.9e-07); F38 other mood [affective] disorders (β = 0.41, p = 0.001); F41, other (non-phobic) anxiety disorders (β = 0.06, p =0.03); F43, reaction to severe stress, and adjustment disorders (β = 0.08, p = 0.004); F60, specific personality disorders (β = 0.09, p = 0.0005); F61, mixed personality disorders (β = 0.18, p = 0.03); F70, mild mental retardation (β = 0.14, p = 0.02) and F79, unspecified mental retardation (β = 0.40, p = 0.047). There were no diagnoses with NLR significantly lower than controls. In the model, age was also positively correlated with NLR (β = 0.005, p < 2e-16; Figure 3). Black ethnicity was associated with lower NLR (β = -0.23, p < 2e-16) and White ethnicity with higher NLR (β = 0.09, p = 5.8e-07; Figure 4). Antipsychotic use (β = 0.04, p = 0.003)) and hypnotic use (β = 0.03, p = 0.01) were both associated with higher NLR (see supplementary materials table S1).

Model 2: NLR between diagnoses with elevated NLR

For peer review only - http://bmjopen.bmj.com/site/about/guidelines.xhtml

We found that of the diagnoses elevated in Model 1, patients with a diagnosis of F38, other mood [affective] disorders, had higher NLR ($\beta = 0.27$, $p = 0.04$), whereas all other diagnoses did not differ significantly (see supplementary materials table S2).

Model 3: NLR across adverse clinical outcomes

There was a positive association between mortality and NLR, adjusting for ICD-10 diagnostic group, age, gender, ethnicity, antipsychotic, antidepressant, mood stabiliser and hypnotic medication use, all of which contributed to the model according to AIC score ($\beta = 0.103$, $p = 2.9e\text{-}08$; see supplementary materials table S3).

There was no significant relationship between number of overnight bed stays or number of face-to-face events and NLR.

Discussion

This study suggests that systemic inflammation occurs in several psychiatric diagnoses. In particular, NLR elevation was seen in patients with dementia, alcohol dependence, schizophrenia, bipolar affective disorder, depression, non-phobic anxiety disorders, and mild mental retardation. In those groups with elevated NLR, the only diagnostic group that had significantly higher NLR was F38 "other mood disorders". All other diagnoses with elevated NLR did not significantly differ in terms of NLR level, suggesting that NLR elevation is a non-specific, cross-diagnostic finding. It is possible that patients with elevated NLR have a shared inflammatory aetiology to their different clinical presentations.

For peer review only - http://bmjopen.bmj.com/site/about/guidelines.xhtml

In comparing our findings to published data, one previous study of NLR found that patients with acute schizophrenia had higher NLR than patients with manic phase BD (Ozdin et al, 2017), whereas Mazza and colleagues (2019b) did not find any difference in NLR between patients with bipolar and unipolar depression. Another group reported that NLR was elevated in patients with Alzheimer's disease compared to age-matched patients with MDD and Parkinson's disease (Baykan et al. 2018). We did not find any evidence for elevated NLR in Alzheimer's disease compared to other diagnoses, but we were not able to exclude the possibility that NLR elevations may occur during acute exacerbations of psychosis.

Our results align closely with findings from other inflammatory biomarkers. A recent meta-analysis indicated variation patterns in inflammatory biomarkers IL-6, TNF and CRP were comparable across diagnoses (Pinto et al, 2017). Thus, current evidence suggests that phenomenologically defined diagnostic criteria do not map closely onto biologically-based pathogenic markers such as NLR (Zachar & Kendler, 2017).

In terms of the association of NLR with patient outcomes, we found a significant positive relationship been NLR and mortality but we found no relationship between NLR and 'number of overnight bed stays' or 'number of face-to-face events'. A previous study investigated a similar clinical outcome measure 'number of hospitalisations' and found a positive relationship with NLR in patients with BD (Melo et al, 2019). The study involved a small sample of 80 patients, but the findings were strengthened by the fact the authors employed a longitudinal design. The difference in findings with our study could be due to the cross-diagnostic nature of our study, and the fact that the patients were not all in-patients.

For peer review only - http://bmjopen.bmj.com/site/about/guidelines.xhtml

A positive relationship between mortality and NLR has been indicated across physical health disorders (Afari & Bhat, 2016, Koh et al, 2015, Templeton et al, 2014). As we were not able to adjust for physical health comorbidities, the relationship that we found may reflect an association between systemic inflammation and poor physical health. Alternatively, our findings may reflect an independent positive relationship between NLR and mortality in psychiatric patients. In both BD patients and MDD patients, NLR has been found to be a significant predictor of suicide risk (Ekinci & Ekinci, 2017, Ivkovic et al, 2016), which may account for some of the increased mortality. However, elevated NLR has been found to independently predict mortality, irrespective of age, sex, education, metastatic cancer, liver disease and depression (Isaac et al, 2016) suggesting systemic inflammation may reduce survival through alternative pathological pathways.

Limitations

This study has several limitations. Confounding factors which may modulate NLR such as body mass index (Azab et al, 2014), smoking (Tulgar et al, 2016), diet and exercise (Gialluisi et al, 2019) were not controlled for and so interactions such as a positive correlation between depression and obesity (Luppino et al, 2010, Mannan et al, 2016) may have confounded our results. Likewise, comorbid physical conditions found to modulate NLR, such as cancer or autoimmune disease (Bhat et al, 2013, Celikbilek et al, 2013, Templeton et al, 2014), were neither excluded nor adjusted for.

The control sample represents a non-institutionalised civilian (US) population. These participants were not screened for physical or mental disorders, so the prevalence of these

For peer review only - http://bmjopen.bmj.com/site/about/guidelines.xhtml

conditions in the control sample is unknown. The fact that samples were selected from different countries and blood cell essays were performed in different laboratories reduces the validity of our patient and control comparisons. However, although NLR was elevated in some ICD-10 diagnoses compared to the control group, NLR in patients with other diagnoses did not significantly differ from this control sample, suggesting that the control NLR was suitable as a comparison group in this study.

This study did not employ validated symptom severity measures. 'Number of overnight bed stays' and 'number of face-to-face events' are logical outcome measures of illness severity. These real-world measures increased the clinical transferability of our results, but they may be affected by unknown factors, such as help-seeking attitudes or illness insight.

This sample is representative of a population seeking secondary care services. Results may not be generalisable to patients seeking primary care services. Finally, this is a cross-sectional study, so causal inferences cannot be inferred.

Nonetheless, this is the largest study to date of cross-diagnostic NLR measurements in a psychiatric population and our sample is representative of adult psychiatric patients in South London. Every patient who accessed SLaM services within our time frame and had available blood count data from age 16 years old or above, was included in our sample. This minimised selection biases. Previous studies included only elderly (Baykan et al, 2018) or Caucasian patients (Mazza et al, 2019b) and so were less generalisable. Our sample is diverse in age and ethnicity so demonstrates improved translational potential. This study

For peer review only - http://bmjopen.bmj.com/site/about/guidelines.xhtml

also compares NLR across the broadest range of diagnoses to date and employs the largest sample yet to address these two research aims.

Future Directions

Findings suggest broad transdiagnostic commonalities regarding systemic inflammation. As within-diagnostic differences in inflammation have also been found (Raison & Miller, 2011, Lamers et al, 2013), it appears inflammatory processes are not bound to traditional diagnostic categories. In both depression and schizophrenia, inflammation is thought to contribute to treatment resistance (Mondelli et al, 2015, Lanquillion, Krieg, Bening-Abu-Shach & Vedder, 2000), perhaps contributing to poor primary treatment response rates in psychiatry (Gaynes et al, 2008, Tiihonen et al, 2017).

It may be fruitful for future studies investigating NLR in psychiatric populations to employ a Research Domain Criteria (RDoC) approach (Cuthbert & Insel, 2010). RDoC reconceptualises psychopathology into transdiagnostic domains, presenting a novel classification of mental illnesses, based on dimensions of observable behaviour and neurobiological measures (Morris & Cuthbert, 2012). As our results indicate inflammatory processes are shared across diagnoses, inflammation may play a role in symptom domains shared by these conditions. For example, alterations in serotonergic, dopaminergic and glutamatergic pathways are consistently reported across mood (Ashok et al, 2017, Cowen, 2008, Werner et al, 2010) and psychotic (Guillin et al, 2007, Stone et al, 2007) disorders and have been reported to be mediated by immunological pathways (Miller, 2009, Miller et al, 2013, Müller & Schwarz, 2007). Immune-mediated alterations in serotoninergic and glutamatergic systems may

For peer review only - http://bmjopen.bmj.com/site/about/guidelines.xhtml

result in mood and cognitive symptoms whilst inflammation-induced modifications of dopaminergic systems may result in motivational changes (Capuron & Castanon, 2016).

NLR may be a useful and cost-effective transdiagnostic parameter by which to stratify psychiatric patients on a common pathophysiological basis. Recent years have seen pharmaceutical companies withdraw investments from psychiatry due to 'low probability of success' (Miller, 2010) and nosology has been highlighted as one of the barriers to research progress (Fibiger et al, 2012). Transdiagnostic biological abnormalities may constitute common targets for pharmacological treatment and NLR may be an accessible tool to aid in identification and stratification of these patients in clinical trials.

Conclusions

Systemic inflammation may occur as a transdiagnostic pathological process in a subpopulation of psychiatric patients. NLR may be an effective biomarker to identify these patients who may benefit from adjunctive anti-inflammatory pharmacological treatment.

Author Statement

The study was conceived by JS with further development by AB. Data extraction was by AB and was specified by JS. Literature review, data analysis first draft of the manuscript was by AB with supervision by JS. Both authors contributed to manuscript preparation and approved the final version.

For peer review only - http://bmjopen.bmj.com/site/about/guidelines.xhtml

For peer review only

For peer review only - http://bmjopen.bmj.com/site/about/guidelines.xhtml

References

Afari, M. E., & Bhat, T. (2016). Neutrophil to lymphocyte ratio (NLR) and cardiovascular diseases: an update. *Expert review of cardiovascular Therapy*, *14*(5), 573-577.

Ashok, A. H., Marques, T. R., Jauhar, S., Nour, M. M., Goodwin, G. M., Young, A. H., & Howes, O. D. (2017). The dopamine hypothesis of bipolar affective disorder: the state of the art and implications for treatment. Molecular psychiatry, 22(5), 666.

Azab, B., Camacho-Rivera, M., & Taioli, E. (2014). Average values and racial differences of neutrophil lymphocyte ratio among a nationally representative sample of United States subjects. *PloS one*, *9*(11), e112361.

Baykan, H., Baykan, Ö., Esen, E. C., Tirak, A., Görgülü, S. A., & Karlıdere, T. (2018). Neutrophil-to-lymphocyte ratio as a potential differential diagnostic marker for alzheimer's disease, major depressive disorder, and parkinson's disease. *The Journal of Psychiatry and Neurological Sciences.* 31. 10.5350/DAJPN2018310407.

Bhat, T., Teli, S., Rijal, J., Bhat, H., Raza, M., Khoueiry, G., ... & Costantino, T. (2013). Neutrophil to lymphocyte ratio and cardiovascular diseases: a review. *Expert review of cardiovascular therapy*, *11*(1), 55-59.

Burwell, SM. (2015). Annual update of the HHS poverty guidelines. *Fed Regist*, *80*, 3236-3237.

Çakur, U., Can Tuman, T., & Yıldırım, O. (2015). Increased neutrophil/lymphoctye ratio in patients with bipolar disorder: a preliminary study. *Psychiatria Danubina*, *27*(2), 0-184.

Capuron, L., & Castanon, N. (2016). Role of inflammation in the development of neuropsychiatric symptom domains: evidence and mechanisms. In *Inflammation-Associated Depression: Evidence, Mechanisms and Implications* (pp. 31-44). Springer, Cham.

Celikbilek, M., Dogan, S., Ozbakır, O., Zararsız, G., Kücük, H., Gürsoy, S., ... & Yücesoy, M. (2013). Neutrophil–lymphocyte ratio as a predictor of disease severity in ulcerative colitis. *Journal of clinical laboratory analysis*, *27*(1), 72-76.

For peer review only - http://bmjopen.bmj.com/site/about/guidelines.xhtml

Centers for Disease Control and Prevention: National Center for Health Statistics (2017). National Health and Nutrition Examination Survey 2015-2016. (https://wwwn.cdc.gov/nchs/nhanes/ContinuousNhanes/Default.aspx?BeginYear=2015). Accessed June 2019.

Chandrashekara, S., Mukhtar Ahmad, M., Renuka, P., Anupama, K. R., & Renuka, K. (2017). Characterization of neutrophil-to-lymphocyte ratio as a measure of inflammation in rheumatoid arthritis. *International journal of rheumatic diseases*, *20*(10), 1457-1467.

Cowen, PJ. (2008). Serotonin and depression: pathophysiological mechanism or marketing myth?. *Trends in Pharmacological Sciences*, *29*(9), 433-436.

Cuthbert, B. N., & Insel, T. R. (2010). Toward new approaches to psychotic disorders: the NIMH Research Domain Criteria project.

Ekinci, O., & Ekinci, A. (2017). The connections among suicidal behavior, lipid profile and low-grade inflammation in patients with major depressive disorder: a specific relationship with the neutrophil-to-lymphocyte ratio. *Nordic journal of psychiatry*, *71*(8), 574-580.

Fond, G. B., & Boyer, L. (2018). C-reactive protein as a peripheral biomarker in schizophrenia. An updated systematic review. *Frontiers in psychiatry*, *9*, 392.

Fibiger, HC. (2012). Psychiatry, the pharmaceutical industry, and the road to better therapeutics.

Gaynes, B. N., Rush, A. J., Trivedi, M. H., Wisniewski, S. R., Spencer, D., & Fava, M. (2008). The STAR* D study: treating depression in the real world. *Cleveland Clinic journal of medicine*, *75*(1), 57-66.

Gialluisi, A., Bonaccio, M., Di Castelnuovo, A., Costanzo, S., De Curtis, A., Sarchiapone, M., ... & Moli-Sani Study Investigators. (2019). Lifestyle and biological factors influence the relationship between mental health and low-grade inflammation. *Brain, behavior, and immunity*.

Gibson, P. H., Croal, B. L., Cuthbertson, B. H., Small, G. R., Ifezulike, A. I., Gibson, G., ... & Hillis, G. S. (2007). Preoperative neutrophil-lymphocyte ratio and outcome from coronary artery bypass grafting. *American heart journal*, *154*(5), 995-1002.

For peer review only - http://bmjopen.bmj.com/site/about/guidelines.xhtml

Guillin, O., Abi-Dargham, A., & Laruelle, M. (2007). Neurobiology of dopamine in schizophrenia. *International review of neurobiology*, *78*, 1-39.

Isaac, V., Wu, C. Y., Huang, C. T., Baune, B. T., Tseng, C. L., & McLachlan, C. S. (2016). Elevated neutrophil to lymphocyte ratio predicts mortality in medical inpatients with multiple chronic conditions. *Medicine*, *95*(23).

Ivković, M., Pantović-Stefanović, M., Dunjić-Kostić, B., Jurišić, V., Lačković, M., Totić-Poznanović, S., Jovanović, A. & Damjanović, A. (2016). Neutrophil-to-lymphocyte ratio predicting suicide risk in euthymic patients with bipolar disorder: Moderatory effect of family history. *Comprehensive psychiatry*, *66*, 87-95.

Karageorgiou, V., Milas, G. P., & Michopoulos, I. (2018). Neutrophil-to-lymphocyte ratio in schizophrenia: A systematic review and meta-analysis. *Schizophrenia research*.

Koh, C. H., Bhoo-Pathy, N., Ng, K. L., Jabir, R. S., Tan, G. H., See, M. H., ... & Taib, N. A. (2015). Utility of pre-treatment neutrophil–lymphocyte ratio and platelet–lymphocyte ratio as prognostic factors in breast cancer. *British journal of cancer*, *113*(1), 150.

Lamers, F., Vogelzangs, N., Merikangas, K. R., De Jonge, P., Beekman, A. T. F., & Penninx, B. W. J. H. (2013). Evidence for a differential role of HPA-axis function, inflammation and metabolic syndrome in melancholic versus atypical depression. *Molecular psychiatry*, *18*(6), 692.

Lanquillon, S., Krieg, J. C., Bening-Abu-Shach, U., & Vedder, H. (2000). Cytokine production and treatment response in major depressive disorder. *Neuropsychopharmacology*, *22*(4), 370-379.

Lopez-Morinigo, J. D., Fernandes, A. C., Shetty, H., Ayesa-Arriola, R., Bari, A., Stewart, R., & Dutta, R. (2018). Can risk assessment predict suicide in secondary mental healthcare? Findings from the South London and Maudsley NHS Foundation Trust Biomedical Research Centre (SLaM BRC) Case Register. *Social psychiatry and psychiatric epidemiology*, *53*(11), 1161-1171.

Luppino, F. S., de Wit, L. M., Bouvy, P. F., Stijnen, T., Cuijpers, P., Penninx, B. W., & Zitman, F. G. (2010). Overweight, obesity, and depression: a systematic review and meta-analysis of longitudinal studies. *Archives of general psychiatry*, *67*(3), 220-229.

For peer review only - http://bmjopen.bmj.com/site/about/guidelines.xhtml

Mannan, M., Mamun, A., Doi, S., & Clavarino, A. (2016). Is there a bi-directional relationship between depression and obesity among adult men and women? Systematic review and bias-adjusted meta analysis. *Asian journal of psychiatry*, *21*, 51-66.

Mazza, M. G., Lucchi, S., Tringali, A. G. M., Rossetti, A., Botti, E. R., & Clerici, M. (2018). Neutrophil/lymphocyte ratio and platelet/lymphocyte ratio in mood disorders: A meta-analysis. *Progress in Neuro-Psychopharmacology and Biological Psychiatry*, *84*, 229-236.

Mazza, M. G., Lucchi, S., Rossetti, A., & Clerici, M. (2019a). Neutrophil-lymphocyte ratio, monocyte-lymphocyte ratio and platelet-lymphocyte ratio in non-affective psychosis: A meta-analysis and systematic review. *The World Journal of Biological Psychiatry*, 1-13.

Mazza, M. G., Tringali, A. G. M., Rossetti, A., Botti, R. E., & Clerici, M. (2019b). Cross-sectional study of neutrophil-lymphocyte, platelet-lymphocyte and monocyte-lymphocyte ratios in mood disorders. *General hospital psychiatry*, *58*, 7-12.

Melo, M. C. A., Garcia, R. F., de Araújo, C. F. C., Abreu, R. L. C., de Bruin, P. F. C., & de Bruin, V. M. S. (2019). Clinical significance of neutrophil-lymphocyte and platelet-lymphocyte ratios in bipolar patients: An 18-month prospective study. *Psychiatry research*, *271*, 8-14.

Miller, AH. (2009). Mechanisms of cytokine-induced behavioral changes: Psychoneuroimmunology at the translational interface. *Brain, behavior, and immunity*, *23*(2), 149-158.

Miller, G. (2010). Is pharma running out of brainy ideas?.

Miller, A. H., Haroon, E., Raison, C. L., & Felger, J. C. (2013). Cytokine targets in the brain: impact on neurotransmitters and neurocircuits. *Depression and anxiety*, *30*(4), 297-306.

Mondelli, V., Ciufolini, S., Belvederi Murri, M., Bonaccorso, S., Di Forti, M., Giordano, A., ... & Pariante, C. M. (2015). Cortisol and inflammatory biomarkers predict poor treatment response in first episode psychosis. *Schizophrenia bulletin*, *41*(5), 1162-1170.

Morris, S. E., & Cuthbert, B. N. (2012). Research Domain Criteria: cognitive systems, neural circuits, and dimensions of behavior. *Dialogues in clinical neuroscience*, *14*(1), 29.

For peer review only - http://bmjopen.bmj.com/site/about/guidelines.xhtml

Müller, N., & Schwarz, M. J. (2007). The immune-mediated alteration of serotonin and glutamate: towards an integrated view of depression. *Molecular psychiatry*, *12*(11), 988.

Office for National Statistics. (2009) *Ethnic group, national identity and religion.* (https://www.ons.gov.uk/methodology/classificationsandstandards/measuringequality/ethnicgroupnationalidentityandreligion). Accessed April 2019.

Osimo, E. F., Cardinal, R. N., Jones, P. B., & Khandaker, G. M. (2018). Prevalence and correlates of low-grade systemic inflammation in adult psychiatric inpatients: An electronic health record-based study. *Psychoneuroendocrinology*, *91*, 226–234. doi:10.1016/j.psyneuen.2018.02.031

Özdin, S., Sarisoy, G., & Böke, Ö. (2017). A comparison of the neutrophil-lymphocyte, platelet-lymphocyte and monocyte-lymphocyte ratios in schizophrenia and bipolar disorder patients–a retrospective file review. *Nordic journal of psychiatry*, *71*(7), 509-512.

Pinto, J. V., Moulin, T. C., & Amaral, O. B. (2017). On the transdiagnostic nature of peripheral biomarkers in major psychiatric disorders: a systematic review. *Neuroscience & Biobehavioral Reviews*, *83*, 97-108.

Raison, C. L., & Miller, A. H. (2011). Is depression an inflammatory disorder?. *Current psychiatry reports*, *13*(6), 467-475.

Stewart, R., Soremekun, M., Perera, G., Broadbent, M., Callard, F., Denis, M., ... & Lovestone, S. (2009). The South London and Maudsley NHS foundation trust biomedical research centre (SLAM BRC) case register: development and descriptive data. *BMC psychiatry*, *9*(1), 51.

Stone, J. M., Morrison, P. D., & Pilowsky, L. S. (2007). Glutamate and dopamine dysregulation in schizophrenia—a synthesis and selective review. *Journal of psychopharmacology*, *21*(4), 440-452.

Templeton, A. J., McNamara, M. G., Šeruga, B., Vera-Badillo, F. E., Aneja, P., Ocaña, A., ... & Tannock, I. F. (2014). Prognostic role of neutrophil-to-lymphocyte ratio in solid tumors: a systematic review and meta-analysis. *JNCI: Journal of the National Cancer Institute*, *106*(6).

For peer review only - http://bmjopen.bmj.com/site/about/guidelines.xhtml

Tiihonen, J., Mittendorfer-Rutz, E., Majak, M., Mehtälä, J., Hoti, F., Jedenius, E., ... & Taipale, H. (2017). Real-world effectiveness of antipsychotic treatments in a nationwide cohort of 29 823 patients with schizophrenia. *JAMA psychiatry*, *74*(7), 686-693.

Tulgar, Y. K., Cakar, S., Tulgar, S., Dalkilic, O., Cakiroglu, B., & Uyanik, B. S. (2016). The effect of smoking on neutrophil/lymphocyte and platelet/lymphocyte ratio and platelet indices: a retrospective study. *Eur Rev Med Pharmacol Sci*, *20*(14), 3112-3118.

Wang, Y., Fuentes, H. E., Attar, B. M., Jaiswal, P., & Demetria, M. (2017). Evaluation of the prognostic value of neutrophil to lymphocyte ratio in patients with hypertriglyceridemia-induced acute pancreatitis. *Pancreatology*, *17*(6), 893-897.

Werner, F. M., & Covenas, R. (2010). Classical neurotransmitters and neuropeptides involved in major depression: a review. *International Journal of Neuroscience*, *120*(7), 455-470.

World Health Organization. (1992). *The ICD-10 classification of mental and behavioural disorders: clinical descriptions and diagnostic guidelines*. Geneva: World Health Organization.

Xiao, TS. (2017). Innate immunity and inflammation. *Cellular & molecular immunology*, *14*(1), 1–3. doi:10.1038/cmi.2016.45

Zachar, P., & Kendler, KS. (2017). The philosophy of nosology. *Annual Review of Clinical Psychology*, *13*, 49-71.

Zahorec, R. (2001). Ratio of neutrophil to lymphocyte counts-rapid and simple parameter of systemic inflammation and stress in critically ill. *Bratislavske lekarske listy*, *102*(1), 5-14.

For peer review only - http://bmjopen.bmj.com/site/about/guidelines.xhtml

Tables

Table 1. Distribution of age, ethnicity and gender variables across patient and control samples. (N = number in group, % = relative percentage of total sample*).*

	Patient Sample		Control Sample	
	N	%	N	%
Age Group				
16-19	1201	8.65	350	8.93
20-29	3188	22.95	600	15.31
30-39	2989	21.52	591	15.08
40-49	2759	19.87	619	15.79
50-59	1987	14.31	584	14.90
60-69	878	6.32	537	13.70
70-79	561	4.04	366	9.34
80+	325	2.34	273	6.96
Ethnicity				
Asian	788	5.67	668	17.04
Black	3749	26.99	1187	30.28
Other/mixed	1238	8.91	217	5.54
White	8113	58.43	1848	47.17
Gender				
Male	6539	47.08	1945	49.6
Female	7346	52.9	1975	50.4
Non-specified	3	0.02	-	-

For peer review only - http://bmjopen.bmj.com/site/about/guidelines.xhtml

Figures

Figure 1: Distribution of Neutrophil to Lymphocyte Ratio (NLR) in patient sample

Figure 2: Distribution of Neutrophil to Lymphocyte Ratio (NLR) in control sample

Figure 3: Neutrophil to Lymphocyte Ratio (NLR) (uncorrected) across age groups

Figure 4: Neutrophil to Lymphocyte Ratio (NLR) (uncorrected) across ethnicity

For peer review only

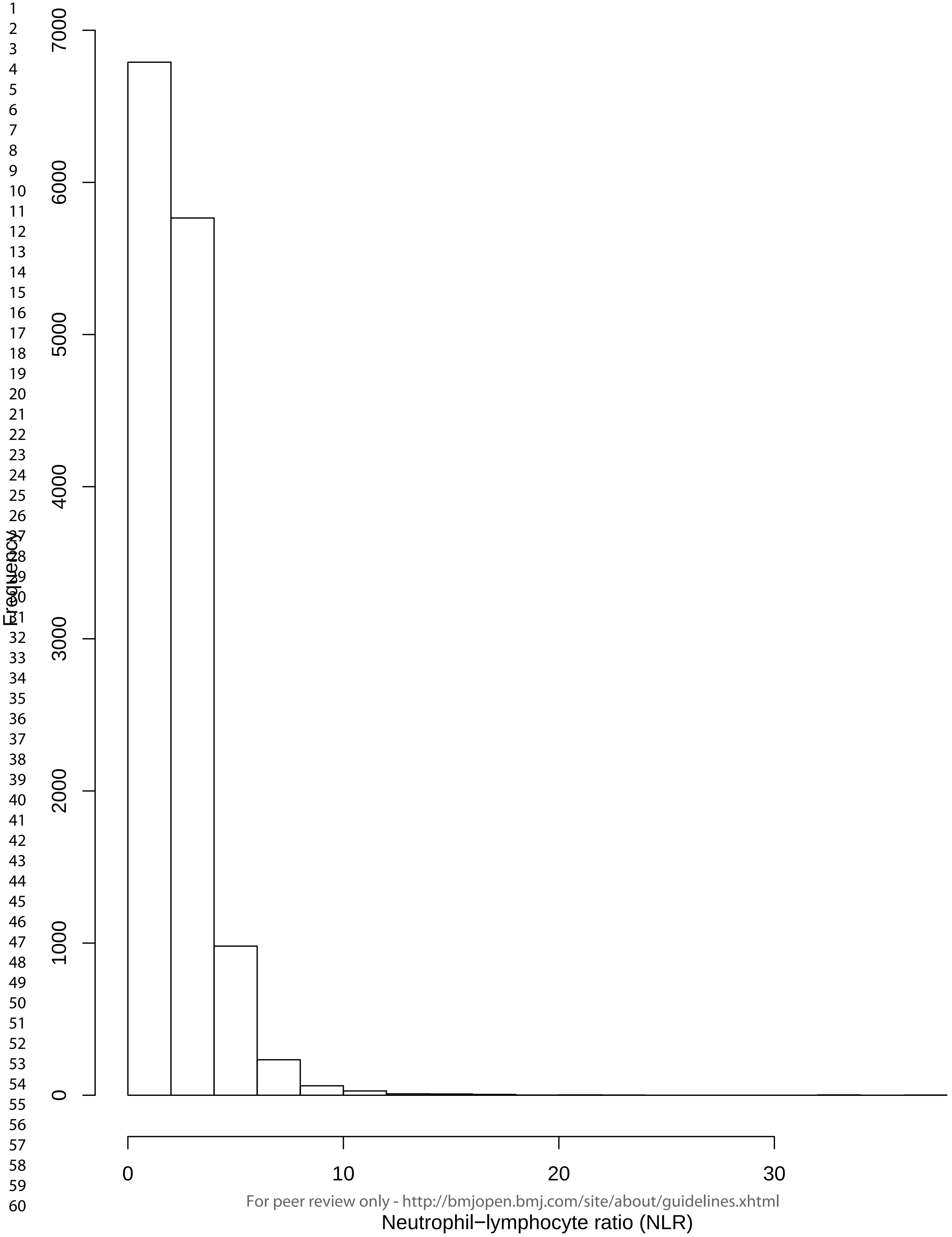
Frequency
0
1000
2000
3000
4000
5000
6000
7000
0
10
20
30
Neutrophil−lymphocyte ratio (NLR)

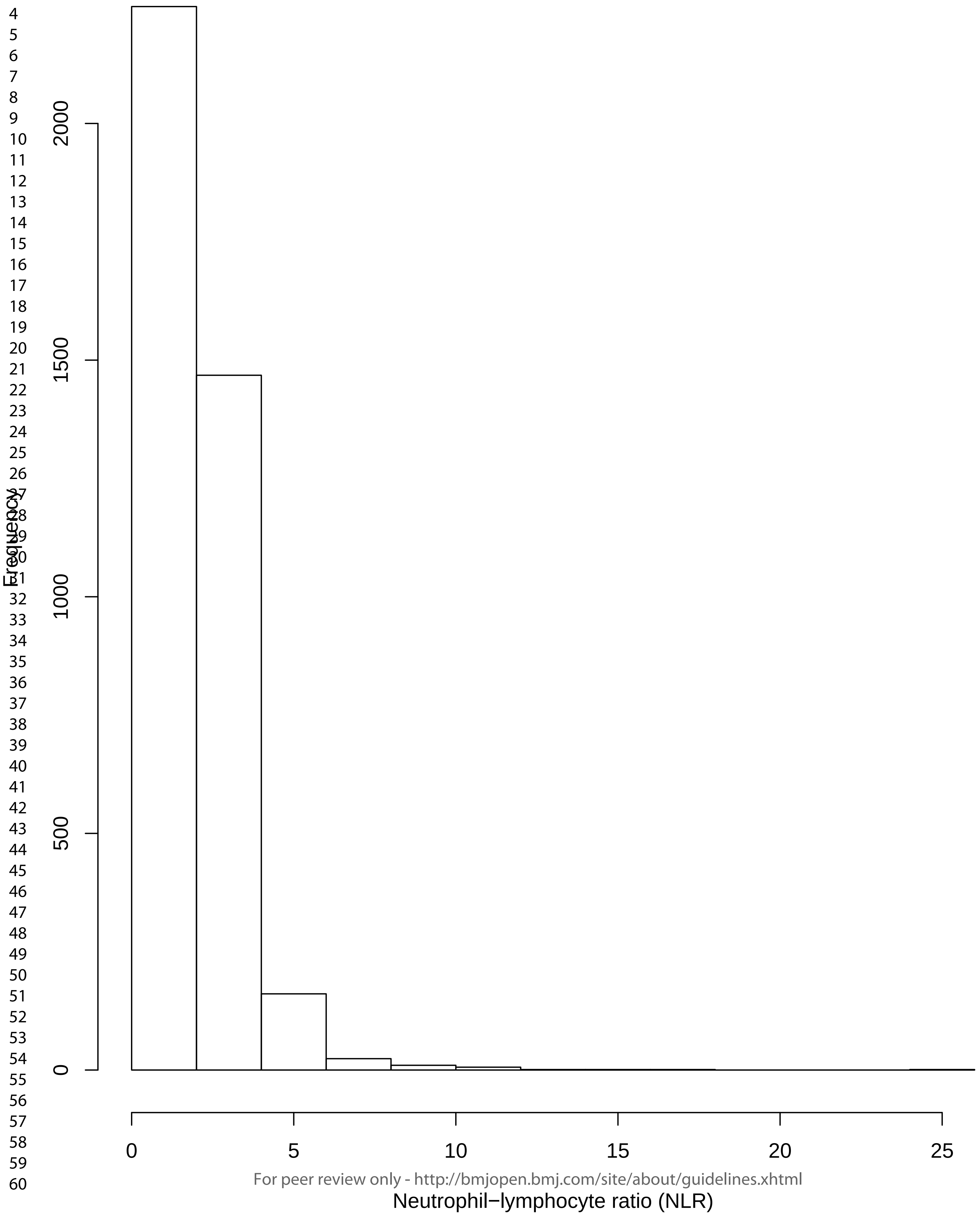
Frequency
0
500
1000
1500
2000
0
5
10
15
20
25
Neutrophil−lymphocyte ratio (NLR)

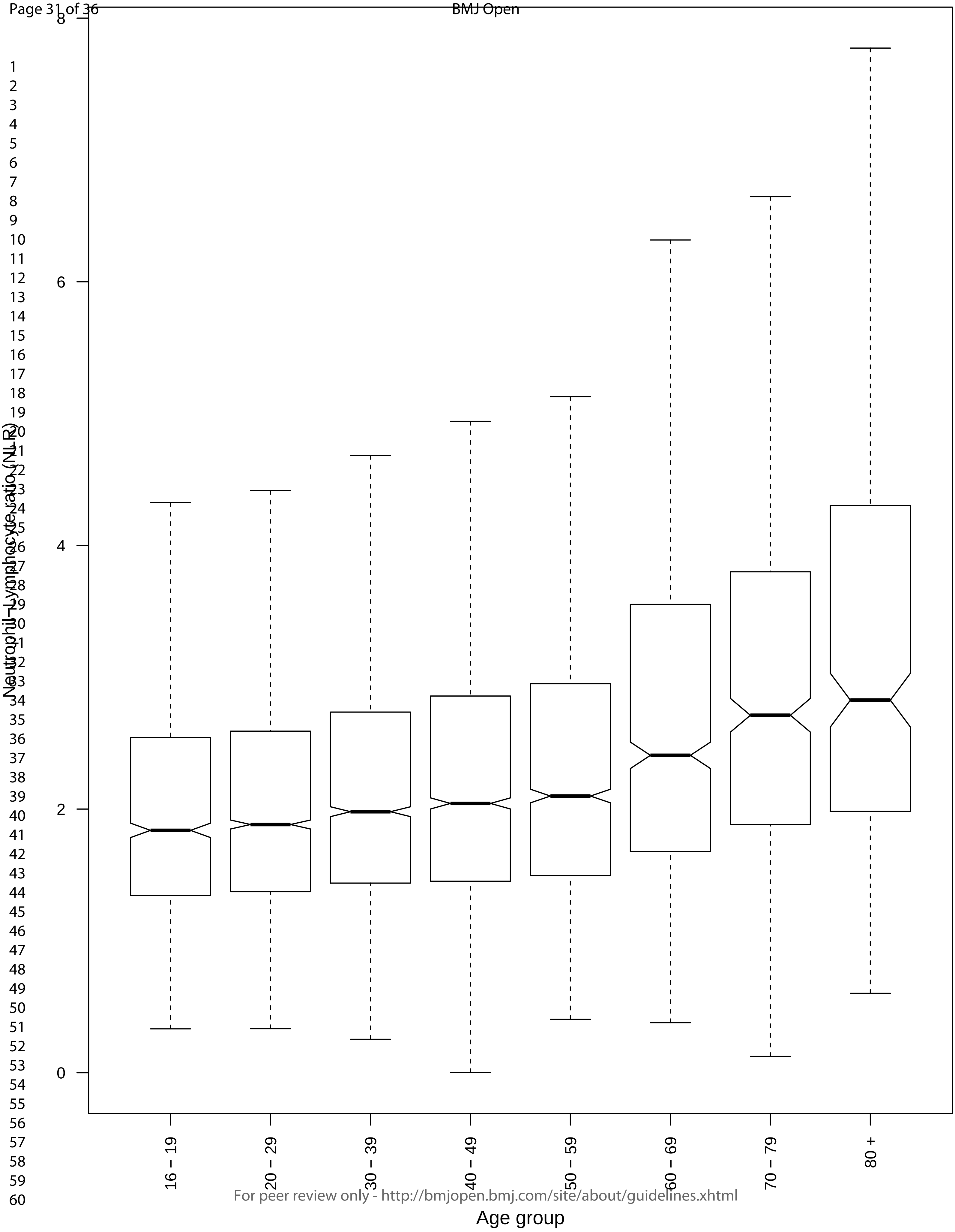
8
6
4
2
0
Neutrophil–Lymphocyte ratio (NLR)
16 – 19
20 – 29
30 – 39
40 – 49
50 – 59
60 – 69
70 – 79
80 +
Age group

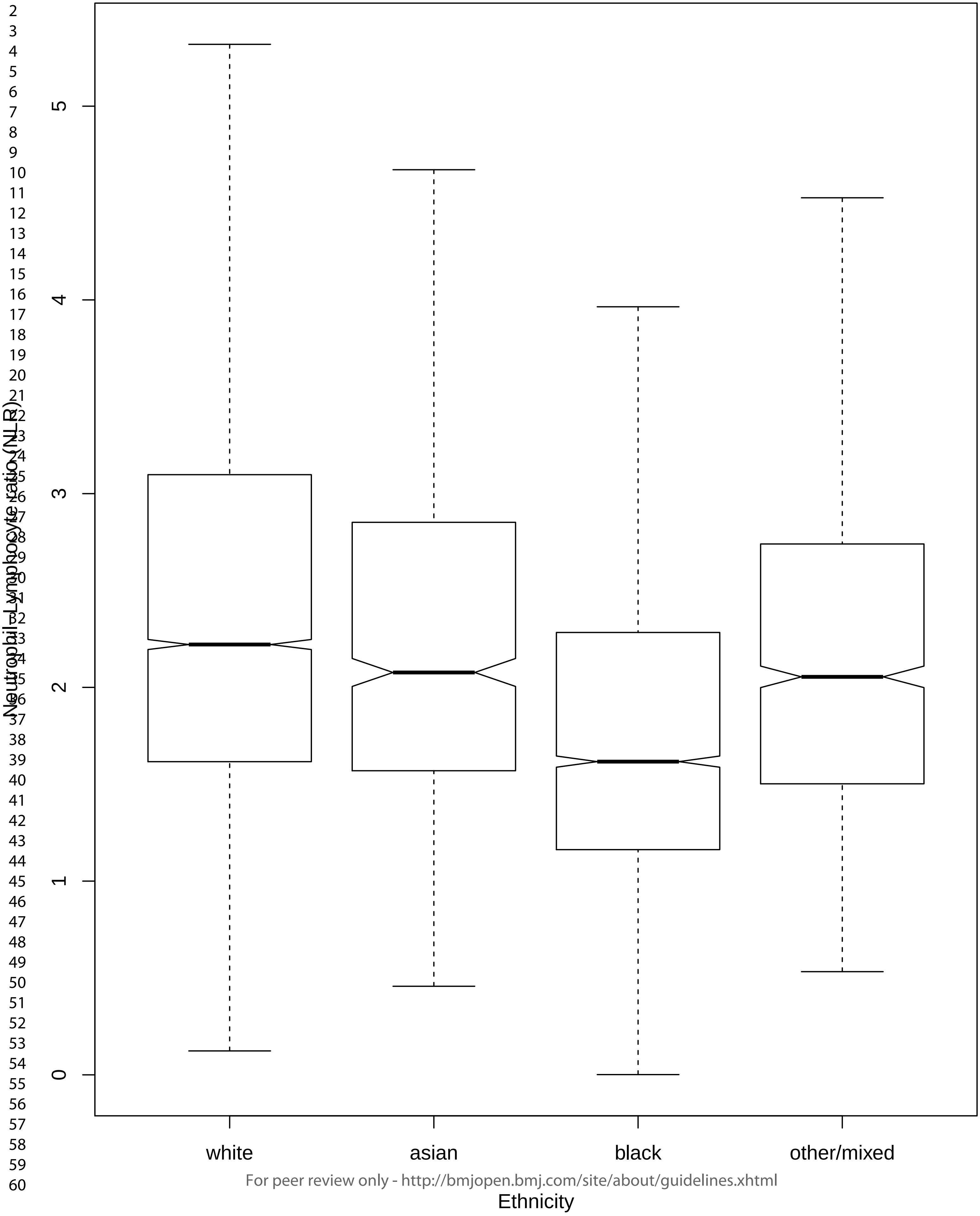
Neutrophil-Lymphocyte ratio (NLR)
0
1
2
3
4
5
white
asian
black
other/mixed
Ethnicity

Supplementary Materials

Table S1: NLR between controls and ICD-10 diagnoses using a log-linked gamma generalised linear model (17737 degrees of freedom).

	Estimate	Std. Error	z value	Pr(>|z|)	
(Intercept)	0.5243	0.023	22.55	< 2e-16	***
F00	0.1483	0.031	4.834	1.34E-06	***
F01	0.2366	0.081	2.922	0.00348	**
F02	0.2687	0.101	2.657	0.00789	**
F03	0.1166	0.064	1.832	0.06699	.
F04	-0.455	0.283	-1.61	0.10796	
F05	0.2782	0.094	2.964	0.00303	**
F06	0.1643	0.048	3.431	0.0006	***
F07	0.1374	0.101	1.362	0.17319	
F09	0.0506	0.108	0.47	0.63829	
F10	0.158	0.021	7.649	2.03E-14	***
F11	0.0185	0.037	0.504	0.614	
F12	0.043	0.05	0.865	0.38709	
F13	0.0085	0.11	0.077	0.93867	
F14	-0.098	0.091	-1.08	0.28035	
F15	-0.159	0.142	-1.12	0.26347	
F16	-0.039	0.2	-0.2	0.84467	
F17	-0.314	0.49	-0.64	0.52183	
F18	-0.112	0.347	-0.32	0.74688	
F19	0.0642	0.036	1.785	0.07423	.
F20	0.0944	0.018	5.331	9.74E-08	***
F21	-0.168	0.186	-0.9	0.36673	
F22	0.0539	0.041	1.308	0.19077	
F23	0.1595	0.029	5.505	3.69E-08	***
F25	0.0737	0.024	3.067	0.00216	**
F28	0.1657	0.076	2.171	0.02992	*
F29	0.1263	0.024	5.207	1.92E-07	***
F30	0.2002	0.043	4.67	3.01E-06	***
F31	0.1161	0.023	5.123	3.01E-07	***
F32	0.1318	0.02	6.514	7.31E-11	***
F33	0.1439	0.029	4.938	7.89E-07	***
F34	-0.113	0.103	-1.1	0.27259	
F38	0.4065	0.127	3.196	0.00139	**
F39	-0.014	0.116	-0.12	0.90559	
F40	-0.137	0.092	-1.49	0.13529	
F41	0.0666	0.031	2.135	0.03277	*
F42	-0.017	0.033	-0.51	0.60838	
F43	0.0829	0.029	2.825	0.00473	**
F44	0.0348	0.049	0.716	0.47408	

For peer review only

F45	0.0248	0.063	0.397	0.69152	
F48	-0.047	0.035	-1.36	0.17526	
F50	0.0274	0.016	1.747	0.08069	.
F52	-0.206	0.2	-1.03	0.30387	
F53	0.0962	0.056	1.728	0.084	.
F55	-0.093	0.49	-0.19	0.84988	
F60	0.0883	0.025	3.476	0.00051	***
F61	0.1759	0.083	2.127	0.03343	*
F64	-0.105	0.283	-0.37	0.7107	
F68	0.1445	0.347	0.417	0.67677	
F69	0.0032	0.186	0.017	0.98645	
F70	0.1351	0.058	2.344	0.01909	*
F71	0.097	0.113	0.855	0.39248	
F72	0.101	0.245	0.412	0.68036	
F78	0.192	0.49	0.392	0.69525	
F79	0.3979	0.201	1.984	0.04731	*
Age	0.005	2E-04	20.22	< 2e-16	***
Black ethnicity	-0.226	0.019	-11.9	< 2e-16	***
Mixed/other ethnicity	-0.022	0.021	-1.07	0.28411	
White ethnicity	0.0921	0.018	4.998	5.79E-07	***
Gender Male	-0.01	0.008	-1.31	0.19134	
Gender Not Specified	-0.425	0.347	-1.22	0.22082	
Gender Other	-0.096	0.49	-0.2	0.84531	
Antipsychotics	0.0375	0.013	2.963	0.00305	**
Antidepressants	0.0115	0.009	1.247	0.21246	
Mood stabilisers	-0.004	0.016	-0.23	0.81925	
Hypnotics	0.025	0.01	2.54	0.01107	*

Signif. codes: ‘***’ 0.001 ‘**’ 0.01 ‘*’ 0.05 ‘.’ 0.1

Table S2: NLR between ICD-10 diagnoses with elevated NLR in first model using a log-linked gamma generalised linear model (10056 degrees of freedom).

	Estimate	Std. Error	z value	Pr(>|z|)	
(Intercept)	0.6081	0.0453	13.412	< 2e-16	***
F01	0.0927	0.0872	1.064	0.28742	
F02	0.1411	0.1075	1.312	0.18937	
F05	0.1351	0.1001	1.351	0.17685	
F06	0.0472	0.0567	0.834	0.40448	
F10	0.0371	0.0359	1.033	0.30155	
F20	-0.0104	0.0337	-0.309	0.75758	
F23	0.0608	0.0423	1.44	0.14991	
F25	-0.0333	0.0376	-0.887	0.37531	

For peer review only

F28	0.0617	0.0841	0.734	0.46269	
F29	0.0288	0.0391	0.736	0.46202	
F30	0.0979	0.0532	1.839	0.06587	.
F31	0.0027	0.0366	0.074	0.9411	
F32	0.0115	0.0357	0.323	0.74672	
F33	0.0154	0.0407	0.378	0.7053	
F38	0.2727	0.1344	2.029	0.0425	*
F41	-0.0555	0.0435	-1.278	0.20121	
F43	-0.027	0.0427	-0.632	0.52728	
F60	-0.0299	0.0395	-0.757	0.44903	
F70	0.0292	0.0662	0.441	0.65913	
F79	0.2722	0.2093	1.301	0.19336	
Age	0.0059	0.0004	16.212	< 2e-16	***
Black ethnicity	-0.2412	0.022	-10.945	< 2e-16	***
Mixed/other ethnicity	-0.0005	0.0262	-0.019	0.98449	
White ethnicity	0.1052	0.0215	4.897	9.73E-07	***
Gender Male	-0.0322	0.0105	-3.069	0.00215	**
Gender Not Specified	-0.1816	0.5072	-0.358	0.72033	
Antipsychotics	0.0331	0.0161	2.055	0.03992	*
Antidepressants	0.0134	0.0113	1.187	0.23507	
Mood stabilisers	-0.0079	0.0168	-0.472	0.63677	
Hypnotics	0.0299	0.0113	2.635	0.00842	**

Signif. codes: '***' 0.001 '**' 0.01 '*' 0.05 '.' 0.1

Table S3: Association between NLR and mortality using a log-linked gamma generalised linear model (13817 degrees of freedom).

	Estimate	Std. Error	z value	Pr(>|z|)	
(Intercept)	0.629	0.042	15.05	< 2e-16	***
Mortality	0.103	0.019	5.547	2.90E-08	***
F01	0.075	0.086	0.881	0.378368	
F02	0.112	0.106	1.06	0.289297	
F03	-0.03	0.069	-0.44	0.662089	
F04	-0.55	0.288	-1.92	0.054981	.
F05	0.123	0.098	1.251	0.211037	
F06	0.048	0.055	0.867	0.385872	
F07	0.01	0.106	0.092	0.9267	
F09	-0.06	0.113	-0.5	0.617792	
F10	0.04	0.035	1.139	0.254815	
F11	-0.1	0.046	-2.19	0.028768	*
F12	-0.05	0.058	-0.93	0.352593	
F13	-0.09	0.115	-0.79	0.427049	

For peer review only

F14	-0.2	0.096	-2.06	0.039388	*
F15	-0.26	0.147	-1.75	0.080089	.
F16	-0.14	0.205	-0.68	0.499768	
F17	-0.43	0.498	-0.86	0.391132	
F18	-0.21	0.353	-0.6	0.5495	
F19	-0.04	0.046	-0.84	0.40397	
F20	-0.01	0.032	-0.3	0.760888	
F21	-0.27	0.19	-1.41	0.159867	
F22	-0.06	0.049	-1.27	0.20328	
F23	0.061	0.041	1.495	0.134955	
F25	-0.03	0.036	-0.86	0.392265	
F28	0.061	0.082	0.747	0.45498	
F29	0.027	0.037	0.709	0.478204	
F30	0.098	0.052	1.895	0.058073	.
F31	0.007	0.035	0.197	0.844041	
F32	0.021	0.035	0.608	0.543242	
F33	0.025	0.04	0.623	0.533192	
F34	-0.22	0.108	-2.07	0.038898	*
F38	0.29	0.132	2.199	0.027893	*
F39	-0.12	0.121	-0.98	0.327305	
F40	-0.24	0.098	-2.47	0.013716	*
F41	-0.04	0.042	-1.04	0.300219	
F42	-0.12	0.044	-2.73	0.006259	**
F43	-0.02	0.041	-0.51	0.611605	
F44	-0.07	0.057	-1.31	0.191077	
F45	-0.09	0.07	-1.25	0.211284	
F48	-0.16	0.046	-3.37	0.000762	***
F50	-0.08	0.035	-2.42	0.015461	*
F52	-0.3	0.205	-1.47	0.141943	
F53	-0.01	0.063	-0.22	0.826974	
F55	-0.19	0.498	-0.39	0.697018	
F60	-0.02	0.038	-0.57	0.571013	
F61	0.076	0.088	0.862	0.388455	
F64	-0.21	0.289	-0.72	0.472579	
F68	0.052	0.352	0.149	0.881745	
F69	-0.1	0.19	-0.53	0.598212	
F70	0.036	0.065	0.559	0.576053	
F71	9E-04	0.118	0.008	0.993735	
F72	0.001	0.25	0.005	0.996367	
F78	0.102	0.498	0.205	0.837852	
F79	0.289	0.205	1.411	0.158226	
Age	0.005	3E-04	16.29	< 2e-16	***
Black ethnicity	-0.24	0.02	-12.1	< 2e-16	***
Mixed/other ethnicity	-0	0.023	-0.16	0.87651	
White ethnicity	0.094	0.019	4.975	6.53E-07	***
Gender Male	-0.03	0.009	-3.14	0.00168	**

For peer review only

Gender Not Specified	-0.42	0.352	-1.2	0.229188	
Gender Other	-0.09	0.497	-0.19	0.853287	
Antipsychotics	0.037	0.013	2.887	0.003889	**
Antidepressants	0.008	0.009	0.88	0.378798	
Mood stabilisers	-0.01	0.016	-0.32	0.748271	
Hypnotics	0.025	0.01	2.479	0.013183	*

Signif. codes: '***' 0.001 '**' 0.01 '*' 0.05 '.' 0.1

For peer review only

BMJ Open

Neutrophil-lymphocyte ratio across psychiatric diagnoses: A cross-sectional study using electronic health records

Journal:	*BMJ Open*
Manuscript ID	bmjopen-2020-036859.R1
Article Type:	Original research
Date Submitted by the Author:	01-May-2020
Complete List of Authors:	Brinn, Aimee; King's College London<br>Stone, James; King's College London,
**Primary Subject Heading**:	Mental health
Secondary Subject Heading:	Mental health
Keywords:	MENTAL HEALTH, Schizophrenia & psychotic disorders < PSYCHIATRY, Depression & mood disorders < PSYCHIATRY, Personality disorders < PSYCHIATRY, Anxiety disorders < PSYCHIATRY

SCHOLARONE™
Manuscripts

I, the Submitting Author has the right to grant and does grant on behalf of all authors of the Work (as defined in the below author licence), an exclusive licence and/or a non-exclusive licence for contributions from authors who are: i) UK Crown employees; ii) where BMJ has agreed a CC-BY licence shall apply, and/or iii) in accordance with the terms applicable for US Federal Government officers or employees acting as part of their official duties; on a worldwide, perpetual, irrevocable, royalty-free basis to BMJ Publishing Group Ltd ("BMJ") its licensees and where the relevant Journal is co-owned by BMJ to the co-owners of the Journal, to publish the Work in this journal and any other BMJ products and to exploit all rights, as set out in our licence.

The Submitting Author accepts and understands that any supply made under these terms is made by BMJ to the Submitting Author unless you are acting as an employee on behalf of your employer or a postgraduate student of an affiliated institution which is paying any applicable article publishing charge ("APC") for Open Access articles. Where the Submitting Author wishes to make the Work available on an Open Access basis (and intends to pay the relevant APC), the terms of reuse of such Open Access shall be governed by a Creative Commons licence – details of these licences and which Creative Commons licence will apply to this Work are set out in our licence referred to above.

Other than as permitted in any relevant BMJ Author's Self Archiving Policies, I confirm this Work has not been accepted for publication elsewhere, is not being considered for publication elsewhere and does not duplicate material already published. I confirm all authors consent to publication of this Work and authorise the granting of this licence.

For peer review only

Neutrophil-lymphocyte ratio across psychiatric diagnoses: A cross-sectional study using electronic health records

Aimee Brinn[1], James M Stone MBBS PhD FRCPsych[1] *

1) King's College London Institute of Psychiatry, Psychology and Neuroscience, De Crespigny Park, London, SE5 8AF

* Address correspondence to: Dr James Stone, Centre for Neuroimaging Sciences, Institute of Psychiatry Psychology & Neuroscience, 16 De Crespigny Park, London SE5 8AF, UK. Email: james.m.stone@kcl.ac.uk.

Keywords: NLR, neutrophil lymphocyte ratio, inflammation, psychiatric diagnoses, mental illness

Abstract word count: 240

Main body word count: 3387

For peer review only

Abstract

Objectives: The main objective of this study was to compare neutrophil-lymphocyte ratio, a marker of systemic inflammation, between patients diagnosed with ICD-10 psychiatric disorders and control participants.

Design: A cross-sectional methodology was employed to retrospectively analyse electronic health records and records derived from a national health survey.

Setting: A secondary mental health care service consisting of four boroughs in South London.

Participants: A diverse sample of 13,888 psychiatric patients extracted from South London and Maudsley electronic health records database and 3,920 control participants extracted from National Health and Nutrition Survey (2015-2016) were included in the study.

Primary and secondary outcome measures: Primary: NLR levels in patients with mental health diagnoses, NLR between patients with different mental health diagnoses. Secondary: Relationship of NLR to length of hospitalisation and to mortality.

Results: NLR was elevated compared to controls in patients with diagnoses including dementia, alcohol dependence, schizophrenia, bipolar affective disorder, depression, non-phobic anxiety disorders, and mild mental retardation ($p < 0.05$). NLR also correlated with age, antipsychotic use and hypnotic use. NLR was found to be higher in individuals of “White” ethnicity and lower in individuals of “Black” ethnicity. Elevated NLR was associated with increased mortality ($\beta = 0.103$, $p = 2.9e-08$) but not with hospital admissions or face-to-face contacts.

Conclusions: Elevated NLR may reflect a transdiagnostic pathological process occurring in a subpopulation of psychiatric patients. NLR may be useful to identify and stratify patients who could benefit from adjunctive anti-inflammatory treatment.

For peer review only - http://bmjopen.bmj.com/site/about/guidelines.xhtml

Article Summary

Strengths and limitations of this study

- Largest study to date of cross-diagnostic neutrophil-lymphocyte measurements in a psychiatric population.
- Sample is representative of diverse adult psychiatric patients in South London.
- Systematic differences between patients and controls reduce the validity of these comparisons.
- This study was retrospective and thus, confounding measures such as body mass index, smoking status and diet were unavailable.

For peer review only

Conflict of Interest

The authors report no conflict of interest.

Financial Support

This paper represents independent research funded by the National Institute for Health Research (NIHR) Biomedical Research Centre at South London and Maudsley NHS Foundation Trust and King's College London. The views expressed are those of the authors and not necessarily those of the NHS, the NIHR or the Department of Health and Social Care.

Data Sharing Statement

Because of their clinical source and under the terms of Ethics and Information Governance approvals, datasets derived from CRIS have to remain within the SLaM firewall. However, all data used for this study will be made accessible on request to cris.administrator@slam.nhs.uk subject to setting up an appropriate SLaM honorary contract or research passport.

For peer review only - http://bmjopen.bmj.com/site/about/guidelines.xhtml

Introduction

Inflammation is a key component of our immune response to physiological injury or infection.[1] Low-grade systemic inflammation is an attenuated, but persistent, form of the inflammatory response and has been found to be prevalent across a range of psychiatric diagnoses, including psychotic, mood, neurotic, and personality, disorders.[2] It has been suggested that neuroinflammation may underlie the pathology of these conditions.[3]

Blood biomarkers are the most commonly used method to study inflammatory processes in psychiatry,[4 5] but many of these are costly or difficult to collect routinely. The neutrophil to lymphocyte ratio (NLR), initially developed as a simple method to assess the level of systemic inflammation in critically ill patients,[6] has more recently been employed to assess systemic inflammation in psychiatric patients.[7 8] NLR can be calculated from a full blood count and is thus cheaper and more readily available than cytokine testing.[9] As it constitutes parameters from both innate (neutrophil) and adaptive (lymphocyte) immune systems, it may be less affected by confounding variables, such as exercise, compared to other commonly used inflammatory biomarkers.[9] NLR has demonstrated reliable prognostic value across a range of physical health disorders,[10-13] highlighting a positive association between systemic inflammation and worse clinical outcomes.

The most studied inflammatory biomarkers in psychiatry, interleukin-6 (IL-6), tumour necrosis factor (TNF) and c-reactive protein (CRP), are elevated in patients with schizophrenia, bipolar disorder and depression, compared to controls.[5] NLR also appears to be elevated in major psychiatric disorders compared to controls.[7 8 14] However, the vast

For peer review only - http://bmjopen.bmj.com/site/about/guidelines.xhtml

proportion of studies have studied a single disorder in comparison to healthy controls, or at best, two different psychiatric disorders.[5] True transdiagnostic studies are rare with only three studies to date comparing NLR between psychiatric diagnoses.[15-17]

Of the NLR studies comparing different psychiatric diagnoses, Ozdin and colleagues reported that patients with schizophrenia, experiencing a psychotic episode, had higher NLR than patients with manic-phase bipolar disorder (BD), suggesting there are detectable differences in NLR across diagnosis and symptomatology.[15] Mazza and colleagues found patients with manic-phase BD had higher NLR than patients with major depressive disorder (MDD).[16] They also found significant differences between manic-phase and depressive-phase BD but not between depressive-phase BD and MDD suggesting that NLR may be elevated in mania but not depression. Lastly, Baykan and colleagues found NLR to be significantly elevated in elderly patients with Alzheimer's (AD), compared to aged-matched patients with MDD and Parkinson's Disease.[17] Shared symptomatology between AD and MDD is problematic for clinicians differentiating between the disorders and the authors suggested that NLR may be an effective diagnostic marker to aid discrimination.

The current evidence base is limited and inconsistent. Further research is required to investigate the role of inflammatory processes in psychiatric illness. Differentially elevated inflammation between different diagnoses may suggest inflammatory processes are more common in specific disorders. Alternatively, comparable elevation between diagnoses may suggest common aetiological pathways, such as shared immunological genetic vulnerabilities and inflammatory processes. Patients with elevated NLR may represent a subtype of psychiatric illness that does not fit within current diagnostic categories. Lastly,

For peer review only - http://bmjopen.bmj.com/site/about/guidelines.xhtml

systemic inflammation may be a by-product of mental health difficulties, reflecting a non-specific comorbid physiological stress.

In this study, we aimed to characterise NLR across a range of ICD-10 psychiatric diagnoses and to investigate the relationship between NLR and adverse clinical outcomes.

Methods

Design

This study employed a cross-sectional methodology to analyse electronic health records extracted using the Clinical Record Interactive Search (CRIS) tool.[18] We retrospectively analysed neutrophil and lymphocyte counts of psychiatric patients using whole blood count data. For comparison, a control group were obtained from the National Health and Nutrition Examination Survey.[19]

Patient and Public Involvement

No patient involvement.

Clinical Record Interactive Search

The Clinical Record Interactive Search (CRIS) application was developed in 2007, with funding from the British National Institute for Health Research. The application generates a data resource consisting of anonymised electronic health records of service users of the SLaM NHS Foundation Trust.[18] These records are contained in the Biomedical Research Centre (BRC) case register, which is updated every 24 hours. CRIS was used to access patient data, using SQL management studio as a platform for data extraction.

For peer review only - http://bmjopen.bmj.com/site/about/guidelines.xhtml

CRIS has ethical approval for usage as an anonymised database for secondary analysis (reference 18/SC/0372). This project was approved by the CRIS Oversight Committee.

Patient Sample

The SLaM BRC case register includes all patients who have accessed secondary mental health care services within a catchment area of four south London boroughs (Lambeth, Southwark, Lewisham and Croydon). SLaM is one of the largest mental healthcare organisations in Europe, providing care to 1.3 million people.[20]

We extracted data from records for all patients who accessed SLaM secondary mental health care between 01-01-2007, when CRIS was established, and 20-11-2018, when our data were extracted.

Data were extracted for all patients meeting the following inclusion criteria:

- ≥ 16 years old at earliest recorded blood test
- ICD-10 Primary Diagnosis F01-F79
- Recorded neutrophil count
- Recorded lymphocyte count

13,888 patients met our inclusion criteria and were included in the sample.

Control Sample

For peer review only - http://bmjopen.bmj.com/site/about/guidelines.xhtml

The control group comprised a sample of participants from the 2015–2016 National Health and Nutrition Survey.[19] The original sample included 9,971 participants from across 30 locations in the United States.. To enable provision of health status indicators for the varied resident population of the United States, the NHANES survey oversamples certain population subgroups. In the 2015-2016 survey cycle, the NHANES oversampled the following subgroups: Hispanic persons, Non-Hispanic black persons, Non-Hispanic Asian persons, persons at or below 185 percent of the Department of Health and Human Services poverty guidelines,[21] and persons aged 80 years and older.

We included individuals meeting the following inclusion criteria:

- ≥16 years old at time of NHANES (2015-2016) survey
- Recorded neutrophil count
- Recorded lymphocyte count

Office for National Statistics (ONS) guidelines do not code 'Hispanic' as a distinct ethnic group.[22] 'Hispanic' was therefore not a distinct ethnic group in the CRIS database. For ease of sample comparison, we excluded Hispanic participants from the NHANES cohort . Following exclusion, 3,920 participants from the NHANES survey met our criteria and were included in the control sample. Any Hispanic participants in the patient group are included by default in the 'Other/Mixed' category.

Variables

Demographics

For peer review only - http://bmjopen.bmj.com/site/about/guidelines.xhtml

Demographic variables were retrieved from structured fields in the CRIS database and included age at earliest blood test, gender and ethnicity. Ethnicity was coded using ONS guidelines.[22] To facilitate comparison with the NHANES sample, we combined 'Other' and 'Mixed' ethnicity codes into one category 'Other/Mixed'.

Neutrophil to lymphocyte ratio

Absolute neutrophil and lymphocyte counts were obtained from routine blood testing data, attained at the point of patient entry to SLaM secondary care services. NLR was calculated for each patient by dividing neutrophil count by lymphocyte count. We extracted earliest available blood count values to minimise potential confounding effects of secondary healthcare treatment.

ICD-10 diagnosis

We included diagnoses from eight ICD-10 diagnostic blocks in this study: Organic, including symptomatic, mental disorders (F01-F09), Mental and behavioural disorders due to psychoactive substance use (F10-F19), Schizophrenia, schizotypal and delusional disorders (F20-F29), mood [affective] disorders (F30-F39), Neurotic, stress-related and somatoform disorders (F40-F49), Behavioural syndromes associated with physiological disturbances and physical factors (F50-59), Disorders of adult personality and behaviour (F60-F69) and Mental retardation (F70-F79). Diagnostic variables were defined by the primary diagnosis assigned to the patient closest to their blood test result.

For peer review only - http://bmjopen.bmj.com/site/about/guidelines.xhtml

We excluded patients with diagnoses in the Disorders of psychological development (F80-F89) and Behavioural/emotional disorders with onset usually occurring in childhood and adolescence (F90-F98) diagnostic blocks as most patients were under 16 years old. We also excluded patients in the Unspecified mental disorder (F99) block due to ambiguity of the diagnosis.

Adverse clinical outcomes

We defined 'number of overnight bed stays' as the total number of overnight hospital stays for each patient since the date of the blood test. Face-to-face events are community face-to-face contacts between a patient and a SLAM-affiliated health professional. This includes patient contact with Community Mental Health Teams, Home Treatment Teams and Liaison A&E; it does not include inpatient contacts. A patient may have multiple face-to-face events per day. The total number of 'face-to-face events' for each patient is measured since the date of the blood test.

Both measures were adjusted for patient time in services since the date of the blood test. Death was recorded as a dichotomous mortality variable.

Medication

The medication prescribed to the patient within 1 year of the blood test date was extracted. Medications were categorised into four classes: antipsychotics, antidepressants, mood stabilisers and hypnotics and were analysed as binary variables.

Statistical Analysis

For peer review only - http://bmjopen.bmj.com/site/about/guidelines.xhtml

We conducted all statistical analyses for this study using R (version 3.6). We analysed data distribution by use of histogram. We report the mean and standard deviation (SD) for normally distributed data.

Because NLR data demonstrated an exponential distribution (see Figures 1 and 2), we conducted regression analyses using a log-linked gamma-family generalised linear model. For each analysis, the shape parameter of the model was estimated using the gamma.shape function from the MASS package, and this was used to set the dispersion parameter as 1/(maximum likelihood estimate). We ran the following models: 1) Control NLR as dependent variable vs. NLR in ICD-10 diagnoses; 2) NLR between all ICD-10 diagnoses that were significantly different from control NLR from Model 1. In Models 1 and 2 age, ethnicity, gender and medication were included as independent variables. We then ran three separate models within the CRIS dataset only to investigate the association of NLR with clinical outcome measures. These models set NLR as the dependent variable vs death, hospital admissions or numbers of face-to face contact. ICD-10 diagnosis, age, ethnicity, gender and medication were included as independent variables. We also ran each model unadjusted for covariates, as recommended by the STROBE statement.[23]

Results

Demographic Results

The mean ages of patients and controls were 40.1 (SD: 16.57) and 46.9 (SD: 19.47) respectively. Due to large sample size and consequent high power of the study to detect small differences, variables were not tested for differences in distribution. Distribution of demographic variables for each group are displayed in Table 1.There was no relationship

For peer review only - http://bmjopen.bmj.com/site/about/guidelines.xhtml

between date of blood test (from which the NLR value was derived) and NLR value across the patient sample (β = -4.76e-06, p = 0.692) suggesting that NLR measurement remained stable across the defined time period (2007-2018).

Model 1: NLR between controls and patients

NLR in the following ICD-10 diagnoses were significantly higher than controls: F00, Alzheimer's disease (β = 0.15, p = 1.3e-06); F01, vascular dementia (β = 0.24, p = 0.003); F02 dementia in other diseases (β = 0.27, p = 0.008); F05, delirium (β =0.28, p = 0.003); F06, other mental disorders due to brain damage and dysfunction (β = 0.16, p = 0.0006); F10, mental and behavioural disorders due to alcohol (β = 0.16, p = 2.0e-14); F20, schizophrenia (β = 0.09, p = 9.7e-08); F23, acute and transient psychotic disorders (β = 0.16, p = 3.7e-08); F25, schizoaffective disorders (β = 0.07, p = 0.002); F28, other nonorganic psychotic disorders (β = 0.17, p = 0.03); F29, unspecified nonorganic psychotic disorders (β = 0.13, p = 1.9e-07); F30, manic episode (β = 0.20, p = 3.0e-06); F31, bipolar affective disorder (β = 0.12, p = 3.0e-07); F32, depressive episode (β = 0.13, p =7.31e-11); F33, recurrent depressive disorder (0.14, p = 7.9e-07); F38 other mood [affective] disorders (β = 0.41, p = 0.001); F41, other (non-phobic) anxiety disorders (β = 0.06, p =0.03); F43, reaction to severe stress, and adjustment disorders (β = 0.08, p = 0.004); F60, specific personality disorders (β = 0.09, p = 0.0005); F61, mixed personality disorders (β = 0.18, p = 0.03); F70, mild mental retardation (β = 0.14, p = 0.02) and F79, unspecified mental retardation (β = 0.40, p = 0.047). NLR in all other diagnoses was not significantly different from controls. In the model, age was positively correlated with NLR (β = 0.005, p < 2e-16; Figure 3). Black ethnicity was associated with lower NLR (β = -0.23, p < 2e-16) and White ethnicity with higher NLR (β = 0.09, p = 5.8e-07; Figure 4). Antipsychotic use (β = 0.04, p = 0.003)) and hypnotic use (β = 0.03, p = 0.01)

For peer review only - http://bmjopen.bmj.com/site/about/guidelines.xhtml

were both associated with higher NLR (see supplementary materials table T1). A secondary analysis unadjusted for covariates revealed a similar pattern of NLR elevation across diagnostic groups compared to controls (see supplementary materials table T2).

Model 2: NLR between diagnoses with elevated NLR

We found that of the diagnoses elevated in Model 1, patients with a diagnosis of F38, other mood [affective] disorders, had higher NLR (β = 0.27, p = 0.04), whereas all other diagnoses did not differ significantly (see supplementary materials table T3). When excluding covariates from the model, we found that many of the diagnostic categories had lower NLR than Alzheimer's disease (see supplementary materials table T4) but given the strong relationship between age and ethnicity on NLR, the significance of this finding is uncertain.

Model 3: NLR across adverse clinical outcomes

There was a positive association between mortality and NLR, both unadjusted (β = 0.274, p = 2e-16) and after correcting for ICD-10 diagnostic group, age, gender, ethnicity, antipsychotic, antidepressant, mood stabiliser and hypnotic medication use (β = 0.103, p = 2.9e-08; see supplementary materials table T5).

There was no significant relationship between number of overnight bed stays or number of face-to-face events and NLR.

Discussion

This study suggests that systemic inflammation occurs in several psychiatric diagnoses. In particular, NLR elevation was seen in patients with dementia, alcohol dependence,

For peer review only - http://bmjopen.bmj.com/site/about/guidelines.xhtml

For peer review only

schizophrenia, bipolar affective disorder, depression, non-phobic anxiety disorders, specific personality disorders, and mild mental retardation. In those groups with elevated NLR, the only diagnostic group that had significantly higher NLR was F38 “other mood disorders”. All other diagnoses with elevated NLR did not significantly differ in terms of NLR level, suggesting that NLR elevation is a non-specific, cross-diagnostic finding. It is possible that patients with elevated NLR have a shared inflammatory aetiology to their different clinical presentations. In contrast, certain diagnoses, including substance misuse (excluding alcohol), eating disorders, and mental retardation (excluding mild mental retardation) did not show significantly elevated NLR from controls after correcting for covariates, suggesting that an inflammatory aetiology is not present in all diagnoses.

The Research Domain Criteria (RDoC) approach reconceptualises psychopathology into transdiagnostic domains,[24] presenting a novel classification of mental illnesses, based on dimensions of observable behaviour and neurobiological measures.[25] As our results indicate inflammatory processes are shared across diagnoses, inflammation may play a role in symptom domains shared by these conditions. For example, alterations in serotonergic, dopaminergic and glutamatergic pathways are consistently reported across mood,[26-28] and psychotic disorders,[29 30] and have been reported to be mediated by immunological pathways.[31-33] Immune-mediated alterations in serotoninergic and glutamatergic systems may result in mood and cognitive symptoms whilst inflammation-induced modifications of dopaminergic systems may result in motivational deficits found across different psychiatric disorders.[34]

For peer review only - http://bmjopen.bmj.com/site/about/guidelines.xhtml

In contrast to previous reports,[15-17] we did not find NLR to differ between bipolar affective disorder and schizophrenia, between bipolar affective disorder or depression, or between depression and Alzheimer's disease after correcting for covariates. This contradiction in findings may be attributed to the larger sample size in our study. Alternatively, NLR may change with acute symptomatology, a hypothesis we were not able to directly test with our dataset.

Our results align closely with findings from other inflammatory biomarkers. A recent meta-analysis indicated variation patterns in inflammatory biomarkers IL-6, TNF and CRP were comparable across diagnoses.[5] Thus, current evidence suggests that phenomenologically defined diagnostic criteria do not map closely onto biologically-based pathogenic markers such as NLR.[35]

Our study also replicated the finding that a positive association exists between NLR and mortality.[36] However, we did not find any relationship between NLR and patient outcomes; there was no relationship between NLR and 'number of overnight bed stays' or 'number of face-to-face events'. This is in contrast to a previous study which found a positive relationship between number of hospitalisations and NLR in patients with BD.[37] The study involved a small sample of 80 patients, but the findings were strengthened by the fact the authors employed a longitudinal design. The difference in findings with our study could be due to the cross-diagnostic nature of our study, and the fact that the patients in our study were not all hospitalised.

For peer review only

A positive relationship between mortality and NLR has been indicated across physical health disorders.[10 12 38] As we were not able to adjust for physical health comorbidities, the relationship that we found may reflect an association between systemic inflammation and poor physical health. Alternatively, our findings may reflect an independent positive relationship between NLR and mortality in psychiatric patients. In both BD patients and MDD patients, NLR has been found to be a significant predictor of suicide risk,[39 40] which may account for some of the increased mortality. However, elevated NLR has been found to independently predict mortality, irrespective of age, sex, education, metastatic cancer, liver disease and depression,[36] suggesting systemic inflammation may reduce survival through alternative pathological pathways.

Limitations

This study has several limitations. Confounding factors which may modulate NLR such as body mass index,[41] smoking,[42] diet and exercise,[43] were not controlled for and so interactions such as a positive correlation between depression and obesity,[44] may have confounded our results. Likewise, comorbid physical conditions found to modulate NLR, such as cancer or autoimmune disease,[38 45 46] or any concurrent infection,[47] were neither excluded nor adjusted for. Moreover, although medications were adjusted for, dosage was not accounted for and this may confound our results. Additionally, only four classes of psychotropic drugs were included in our model; other medications, such as steroid and NSAID drugs, are known to affect systemic inflammation but were neither excluded nor adjusted for.

For peer review only - http://bmjopen.bmj.com/site/about/guidelines.xhtml

The control sample represents a non-institutionalised civilian (US) population. These participants were not screened for physical or mental disorders, so the prevalence of these conditions in the control sample is unknown. The fact that samples were selected from different countries and blood cell essays were performed in different laboratories reduces the validity of our patient and control comparisons. However, although NLR was elevated in some ICD-10 diagnoses compared to the control group, NLR in patients with other diagnoses did not significantly differ from this control sample, suggesting that the control NLR was suitable as a comparison group in this study.

This study did not employ validated symptom severity measures. 'Number of overnight bed stays' and 'number of face-to-face events' are logical outcome measures of illness severity. These real-world measures increased the clinical transferability of our results, but they may be affected by unknown factors, such as help-seeking attitudes or illness insight.

This sample is representative of a population seeking secondary care services. Results may not be generalisable to patients seeking primary care services. Finally, this is a cross-sectional study based on a single measurement of NLR for each patient. The one measurement may not reflect inflammatory status over time and causal inferences cannot be made.

Nonetheless, this is the largest study to date of cross-diagnostic NLR measurements in a psychiatric population and our sample is representative of adult psychiatric patients in South London. Every patient who accessed SLaM services within our time frame and had available blood count data from age 16 years old or above, was included in our sample. This

For peer review only - http://bmjopen.bmj.com/site/about/guidelines.xhtml

minimised selection biases. Previous studies included only elderly,[17] or Caucasian patients,[16] and so were less generalisable. Our sample is diverse in age and ethnicity and so demonstrates improved translational potential. This study also compares NLR across the broadest range of diagnoses to date and employs the largest sample yet to address these two research aims.

Future Directions

Findings suggest broad transdiagnostic commonalities regarding systemic inflammation. As within-diagnostic differences in inflammation have also been found,[48 49] it appears inflammatory processes are not bound to traditional diagnostic categories. In both depression and schizophrenia, inflammation is thought to contribute to treatment resistance,[50 51] perhaps contributing to poor primary treatment response rates in psychiatry.[52 53]

It may be fruitful for future studies investigating NLR in psychiatric populations to employ a Research Domain Criteria (RDoC) approach.[24] NLR may be a cost-effective and efficient biomarker by which to stratify psychiatric patients on a common pathophysiological basis. Conducting research without the restraints of nosology may elucidate transdiagnostic symptom domains associated with systemic inflammation. Recent years have seen pharmaceutical companies withdraw investments from psychiatry due to 'low probability of success',[54] and nosology has been highlighted as one of the barriers to research progress.[55] Transdiagnostic biological abnormalities may constitute common targets for pharmacological treatment and NLR may be an accessible tool to aid in identification and stratification of these patients in clinical trials.

For peer review only - http://bmjopen.bmj.com/site/about/guidelines.xhtml

Lastly, future research should aim to measure NLR levels prior to and following successful treatment; changes in NLR post-treatment may indicate that increased NLR levels are associated with acute symptomatology, rather than inherent vulnerability.

Conclusions

Systemic inflammation may occur as a transdiagnostic pathological process in a subpopulation of psychiatric patients. NLR may be an effective biomarker to identify these patients who may benefit from adjunctive anti-inflammatory pharmacological treatment.

Author Statement

The study was proposed by JS. This proposal provided an outline of study objectives, methods and statistical analyses. Data extraction was specified by JS and completed by AB. Supervised by JS, AB conducted a literature review which informed further development of the study design, such as indicating which confounding variables were to be included in the regression model. AB completed the abstract, introduction, methods, data analysis, results and discussion for the first draft of the manuscript. JS provided guidance throughout with specific instruction for the data analysis and results sections. Both authors contributed to the final manuscript and approved the final version.

For peer review only - http://bmjopen.bmj.com/site/about/guidelines.xhtml

Tables

Table 1. Distribution of age, ethnicity and gender variables across patient and control samples. (N = number in group, % = relative percentage of total sample*).*

	Patient Sample		Control Sample	
	N	%	N	%
Age Group				
16-19	1201	8.65	350	8.93
20-29	3188	22.95	600	15.31
30-39	2989	21.52	591	15.08
40-49	2759	19.87	619	15.79
50-59	1987	14.31	584	14.90
60-69	878	6.32	537	13.70
70-79	561	4.04	366	9.34
80+	325	2.34	273	6.96
Ethnicity				
Asian	788	5.67	668	17.04
Black	3749	26.99	1187	30.28
Other/mixed	1238	8.91	217	5.54
White	8113	58.43	1848	47.17
Gender				
Male	6539	47.08	1945	49.6
Female	7346	52.9	1975	50.4
Non-specified	3	0.02	-	-

For peer review only - http://bmjopen.bmj.com/site/about/guidelines.xhtml

Figures

Figure 1: Distribution of Neutrophil to Lymphocyte Ratio (NLR) in patient sample

Figure 2: Distribution of Neutrophil to Lymphocyte Ratio (NLR) in control sample

Figure 3: Neutrophil to Lymphocyte Ratio (NLR) (uncorrected) across ethnicity

Figure 4: Neutrophil to Lymphocyte Ratio (NLR) (uncorrected) across age groups

For peer review only

References

1. Xiao TS. Innate immunity and inflammation. *Cell Mol Immunol* 2017;14(1):1-3. doi: 10.1038/cmi.2016.45 [published Online First: 2016/08/23]
2. Osimo EF, Cardinal RN, Jones PB, et al. Prevalence and correlates of low-grade systemic inflammation in adult psychiatric inpatients: An electronic health record-based study. *Psychoneuroendocrinology* 2018;91:226-34. doi: 10.1016/j.psyneuen.2018.02.031 [published Online First: 2018/03/17]
3. Najjar S, Pearlman DM, Alper K, et al. Neuroinflammation and psychiatric illness. *J Neuroinflammation* 2013;10:43. doi: 10.1186/1742-2094-10-43 [published Online First: 2013/04/04]
4. Fond G, Lancon C, Auquier P, et al. C-Reactive Protein as a Peripheral Biomarker in Schizophrenia. An Updated Systematic Review. *Front Psychiatry* 2018;9:392. doi: 10.3389/fpsyt.2018.00392 [published Online First: 2018/09/08]
5. Pinto JV, Moulin TC, Amaral OB. On the transdiagnostic nature of peripheral biomarkers in major psychiatric disorders: A systematic review. *Neurosci Biobehav Rev* 2017;83:97-108. doi: 10.1016/j.neubiorev.2017.10.001 [published Online First: 2017/10/08]
6. Zahorec R. Ratio of neutrophil to lymphocyte counts--rapid and simple parameter of systemic inflammation and stress in critically ill. *Bratisl Lek Listy* 2001;102(1):5-14. [published Online First: 2001/11/29]
7. Karageorgiou V, Milas GP, Michopoulos I. Neutrophil-to-lymphocyte ratio in schizophrenia: A systematic review and meta-analysis. *Schizophr Res* 2019;206:4-12. doi: 10.1016/j.schres.2018.12.017 [published Online First: 2018/12/24]
8. Mazza MG, Lucchi S, Tringali AGM, et al. Neutrophil/lymphocyte ratio and platelet/lymphocyte ratio in mood disorders: A meta-analysis. *Prog Neuropsychopharmacol Biol Psychiatry* 2018;84(Pt A):229-36. doi: 10.1016/j.pnpbp.2018.03.012 [published Online First: 2018/03/15]
9. Gibson PH, Croal BL, Cuthbertson BH, et al. Preoperative neutrophil-lymphocyte ratio and outcome from coronary artery bypass grafting. *Am Heart J* 2007;154(5):995-1002. doi: 10.1016/j.ahj.2007.06.043 [published Online First: 2007/10/31]
10. Afari ME, Bhat T. Neutrophil to lymphocyte ratio (NLR) and cardiovascular diseases: an update. *Expert Rev Cardiovasc Ther* 2016;14(5):573-7. doi: 10.1586/14779072.2016.1154788 [published Online First: 2016/02/16]
11. Chandrashekara S, Mukhtar Ahmad M, Renuka P, et al. Characterization of neutrophil-to-lymphocyte ratio as a measure of inflammation in rheumatoid arthritis. *Int J Rheum Dis* 2017;20(10):1457-67. doi: 10.1111/1756-185X.13157 [published Online First: 2017/09/28]
12. Koh CH, Bhoo-Pathy N, Ng KL, et al. Utility of pre-treatment neutrophil-lymphocyte ratio and platelet-lymphocyte ratio as prognostic factors in breast cancer. *Br J Cancer* 2015;113(1):150-8. doi: 10.1038/bjc.2015.183 [published Online First: 2015/05/30]
13. Wang Y, Fuentes HE, Attar BM, et al. Evaluation of the prognostic value of neutrophil to lymphocyte ratio in patients with hypertriglyceridemia-induced acute pancreatitis. *Pancreatology* 2017;17(6):893-97. doi: 10.1016/j.pan.2017.10.001 [published Online First: 2017/10/17]
14. Mazza MG, Lucchi S, Rossetti A, et al. Neutrophil-lymphocyte ratio, monocyte-lymphocyte ratio and platelet-lymphocyte ratio in non-affective psychosis: A meta-analysis and systematic review. *World J Biol Psychiatry* 2019:1-13. doi: 10.1080/15622975.2019.1583371 [published Online First: 2019/02/27]
15. Ozdin S, Sarisoy G, Boke O. A comparison of the neutrophil-lymphocyte, platelet-lymphocyte and monocyte-lymphocyte ratios in schizophrenia and bipolar disorder patients - a retrospective file review. *Nord J Psychiatry* 2017;71(7):509-12. doi: 10.1080/08039488.2017.1340517 [published Online First: 2017/06/24]

For peer review only - http://bmjopen.bmj.com/site/about/guidelines.xhtml

16. Mazza MG, Tringali AGM, Rossetti A, et al. Cross-sectional study of neutrophil-lymphocyte, platelet-lymphocyte and monocyte-lymphocyte ratios in mood disorders. *Gen Hosp Psychiatry* 2019;58:7-12. doi: 10.1016/j.genhosppsych.2019.02.003 [published Online First: 2019/03/01]
17. Baykan H, Baykan Ö, Esen EC, et al. Neutrophil-to-Lymphocyte Ratio as a Potential Differential Diagnostic Marker for Alzheimer's Disease, Major Depressive Disorder, and Parkinson's Disease. *J Psych Neurol Sci* 2018 doi: 10.5350/DAJPN2018310407
18. Stewart R, Soremekun M, Perera G, et al. The South London and Maudsley NHS Foundation Trust Biomedical Research Centre (SLAM BRC) case register: development and descriptive data. *BMC Psychiatry* 2009;9:51. doi: 10.1186/1471-244X-9-51 [published Online First: 2009/08/14]
19. Statistics CfDCaPNCfH. National Health and Nutrition Examination Survey 2015-2016. . 2017
20. Lopez-Morinigo JD, Fernandes AC, Shetty H, et al. Can risk assessment predict suicide in secondary mental healthcare? Findings from the South London and Maudsley NHS Foundation Trust Biomedical Research Centre (SLaM BRC) Case Register. *Soc Psychiatry Psychiatr Epidemiol* 2018;53(11):1161-71. doi: 10.1007/s00127-018-1536-8 [published Online First: 2018/06/04]
21. Burwell SM. Annual update of the HHS poverty guidelines. *Fed Regist* 2015;80:3236-37.
22. Statistics OfN. Ethnic group, national identity and religion. 2009
23. Vandenbroucke JP, von Elm E, Altman DG, et al. Strengthening the Reporting of Observational Studies in Epidemiology (STROBE): explanation and elaboration. *PLoS Med* 2007;4(10):e297. doi: 10.1371/journal.pmed.0040297 [published Online First: 2007/10/19]
24. Cuthbert BN, Insel TR. Toward new approaches to psychotic disorders: the NIMH Research Domain Criteria project. *Schizophr Bull* 2010;36(6):1061-2. doi: 10.1093/schbul/sbq108 [published Online First: 2010/10/12]
25. Morris SE, Cuthbert BN. Research Domain Criteria: cognitive systems, neural circuits, and dimensions of behavior. *Dialogues Clin Neurosci* 2012;14(1):29-37. [published Online First: 2012/05/12]
26. Ashok AH, Marques TR, Jauhar S, et al. The dopamine hypothesis of bipolar affective disorder: the state of the art and implications for treatment. *Mol Psychiatry* 2017;22(5):666-79. doi: 10.1038/mp.2017.16 [published Online First: 2017/03/16]
27. Cowen PJ. Serotonin and depression: pathophysiological mechanism or marketing myth? *Trends Pharmacol Sci* 2008;29(9):433-6. doi: 10.1016/j.tips.2008.05.004 [published Online First: 2008/07/01]
28. Werner FM, Covenas R. Classical neurotransmitters and neuropeptides involved in major depression: a review. *Int J Neurosci* 2010;120(7):455-70. doi: 10.3109/00207454.2010.483651 [published Online First: 2010/06/30]
29. Guillin O, Abi-Dargham A, Laruelle M. Neurobiology of dopamine in schizophrenia. *Int Rev Neurobiol* 2007;78:1-39. doi: 10.1016/S0074-7742(06)78001-1 [published Online First: 2007/03/14]
30. Stone JM, Morrison PD, Pilowsky LS. Glutamate and dopamine dysregulation in schizophrenia--a synthesis and selective review. *J Psychopharmacol* 2007;21(4):440-52. doi: 10.1177/0269881106073126 [published Online First: 2007/01/30]
31. Miller AH. Mechanisms of cytokine-induced behavioral changes: Psychoneuroimmunology at the translational interface. *Brain, Behavior, and Immunity* 2009;23(2):149-58.
32. Miller AH, Haroon E, Raison CL, et al. Cytokine targets in the brain: impact on neurotransmitters and neurocircuits. *Depress Anxiety* 2013;30(4):297-306. doi: 10.1002/da.22084 [published Online First: 2013/03/08]
33. Muller N, Schwarz MJ. The immune-mediated alteration of serotonin and glutamate: towards an integrated view of depression. *Mol Psychiatry* 2007;12(11):988-1000. doi: 10.1038/sj.mp.4002006 [published Online First: 2007/04/26]

For peer review only - http://bmjopen.bmj.com/site/about/guidelines.xhtml

34. Capuron L, Castanon N. Role of Inflammation in the Development of Neuropsychiatric Symptom Domains: Evidence and Mechanisms. *Curr Top Behav Neurosci* 2017;31:31-44. doi: 10.1007/7854_2016_14 [published Online First: 2016/05/26]
35. Zachar P, Kendler KS. The Philosophy of Nosology. *Annu Rev Clin Psychol* 2017;13:49-71. doi: 10.1146/annurev-clinpsy-032816-045020 [published Online First: 2017/05/10]
36. Isaac V, Wu CY, Huang CT, et al. Elevated neutrophil to lymphocyte ratio predicts mortality in medical inpatients with multiple chronic conditions. *Medicine (Baltimore)* 2016;95(23):e3832. doi: 10.1097/MD.0000000000003832 [published Online First: 2016/06/10]
37. Melo MCA, Garcia RF, de Araujo CFC, et al. Clinical significance of neutrophil-lymphocyte and platelet-lymphocyte ratios in bipolar patients: An 18-month prospective study. *Psychiatry Res* 2019;271:8-14. doi: 10.1016/j.psychres.2018.10.077 [published Online First: 2018/11/19]
38. Templeton AJ, McNamara MG, Seruga B, et al. Prognostic role of neutrophil-to-lymphocyte ratio in solid tumors: a systematic review and meta-analysis. *J Natl Cancer Inst* 2014;106(6):dju124. doi: 10.1093/jnci/dju124 [published Online First: 2014/05/31]
39. Ekinci O, Ekinci A. The connections among suicidal behavior, lipid profile and low-grade inflammation in patients with major depressive disorder: a specific relationship with the neutrophil-to-lymphocyte ratio. *Nord J Psychiatry* 2017;71(8):574-80. doi: 10.1080/08039488.2017.1363285 [published Online First: 2017/08/12]
40. Ivkovic M, Pantovic-Stefanovic M, Dunjic-Kostic B, et al. Neutrophil-to-lymphocyte ratio predicting suicide risk in euthymic patients with bipolar disorder: Moderatory effect of family history. *Compr Psychiatry* 2016;66:87-95. doi: 10.1016/j.comppsych.2016.01.005 [published Online First: 2016/03/21]
41. Azab B, Camacho-Rivera M, Taioli E. Average values and racial differences of neutrophil lymphocyte ratio among a nationally representative sample of United States subjects. *PLoS One* 2014;9(11):e112361. doi: 10.1371/journal.pone.0112361 [published Online First: 2014/11/07]
42. Tulgar YK, Cakar S, Tulgar S, et al. The effect of smoking on neutrophil/lymphocyte and platelet/lymphocyte ratio and platelet indices: a retrospective study. *Eur Rev Med Pharmacol Sci* 2016;20(14):3112-8. [published Online First: 2016/07/28]
43. Gialluisi A, Bonaccio M, Di Castelnuovo A, et al. Lifestyle and biological factors influence the relationship between mental health and low-grade inflammation. *Brain Behav Immun* 2020;85:4-13. doi: 10.1016/j.bbi.2019.04.041 [published Online First: 2019/05/06]
44. Luppino FS, de Wit LM, Bouvy PF, et al. Overweight, obesity, and depression: a systematic review and meta-analysis of longitudinal studies. *Arch Gen Psychiatry* 2010;67(3):220-9. doi: 10.1001/archgenpsychiatry.2010.2 [published Online First: 2010/03/03]
45. Bhat T, Teli S, Rijal J, et al. Neutrophil to lymphocyte ratio and cardiovascular diseases: a review. *Expert Rev Cardiovasc Ther* 2013;11(1):55-9. doi: 10.1586/erc.12.159 [published Online First: 2012/12/25]
46. Celikbilek M, Dogan S, Ozbakir O, et al. Neutrophil-lymphocyte ratio as a predictor of disease severity in ulcerative colitis. *J Clin Lab Anal* 2013;27(1):72-6. doi: 10.1002/jcla.21564 [published Online First: 2013/01/08]
47. Kim HA, Jung JY, Suh CH. Usefulness of neutrophil-to-lymphocyte ratio as a biomarker for diagnosing infections in patients with systemic lupus erythematosus. *Clin Rheumatol* 2017;36(11):2479-85. doi: 10.1007/s10067-017-3792-5 [published Online First: 2017/08/26]
48. Raison CL, Miller AH. Is depression an inflammatory disorder? *Curr Psychiatry Rep* 2011;13(6):467-75. doi: 10.1007/s11920-011-0232-0 [published Online First: 2011/09/20]
49. Lamers F, Vogelzangs N, Merikangas KR, et al. Evidence for a differential role of HPA-axis function, inflammation and metabolic syndrome in melancholic versus atypical depression.

For peer review only - http://bmjopen.bmj.com/site/about/guidelines.xhtml

Mol Psychiatry 2013;18(6):692-9. doi: 10.1038/mp.2012.144 [published Online First: 2012/10/24]
50. Mondelli V, Ciufolini S, Belvederi Murri M, et al. Cortisol and Inflammatory Biomarkers Predict Poor Treatment Response in First Episode Psychosis. *Schizophr Bull* 2015;41(5):1162-70. doi: 10.1093/schbul/sbv028 [published Online First: 2015/04/02]
51. Lanquillon S, Krieg JC, Bening-Abu-Shach U, et al. Cytokine production and treatment response in major depressive disorder. *Neuropsychopharmacology* 2000;22(4):370-9. doi: 10.1016/S0893-133X(99)00134-7 [published Online First: 2000/03/04]
52. Gaynes BN, Rush AJ, Trivedi MH, et al. The STAR*D study: treating depression in the real world. *Cleve Clin J Med* 2008;75(1):57-66. doi: 10.3949/ccjm.75.1.57 [published Online First: 2008/02/02]
53. Tiihonen J, Mittendorfer-Rutz E, Majak M, et al. Real-World Effectiveness of Antipsychotic Treatments in a Nationwide Cohort of 29823 Patients With Schizophrenia. *JAMA Psychiatry* 2017;74(7):686-93. doi: 10.1001/jamapsychiatry.2017.1322 [published Online First: 2017/06/09]
54. Miller G. Is Pharma Running Out of Brainy Ideas? *Science* 2010 doi: 10.1126/science.329.5991.502
55. Fibiger HC. Psychiatry, the pharmaceutical industry, and the road to better therapeutics. *Schizophr Bull* 2012;38(4):649-50. doi: 10.1093/schbul/sbs073 [published Online First: 2012/07/28]

For peer review only

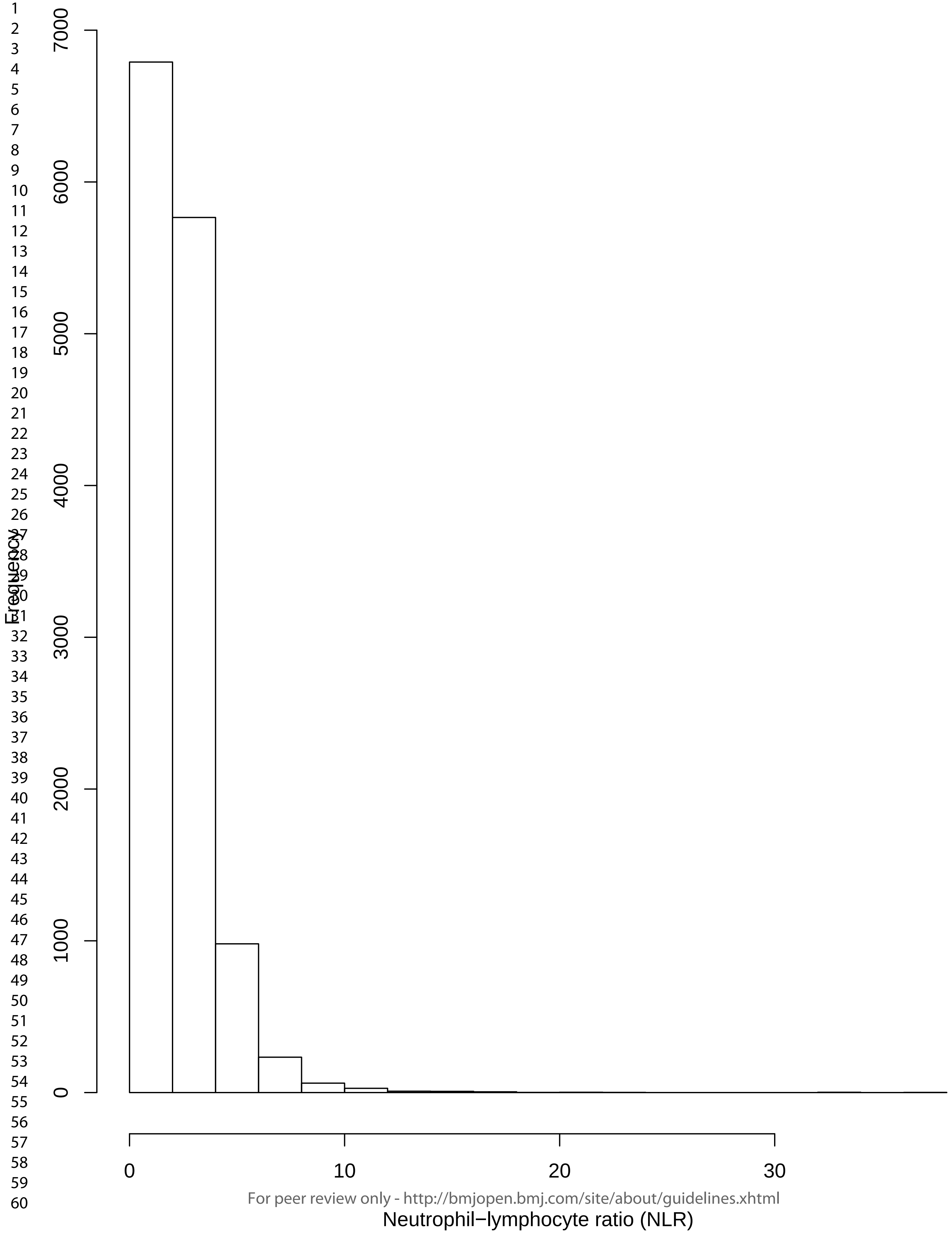

Frequency
0
1000
2000
3000
4000
5000
6000
7000
0
10
20
30
Neutrophil−lymphocyte ratio (NLR)

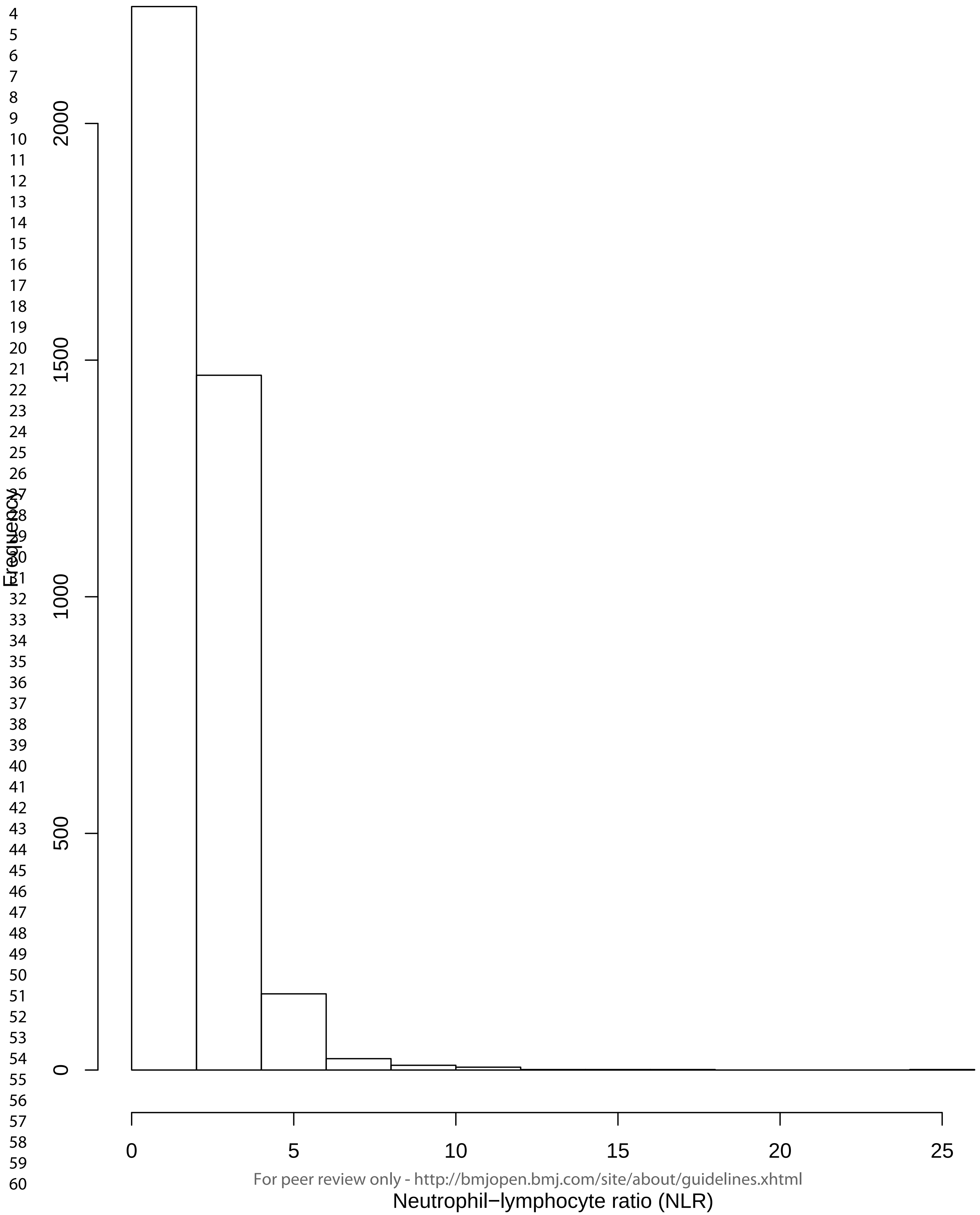

For peer review only - http://bmjopen.bmj.com/site/about/guidelines.xhtml

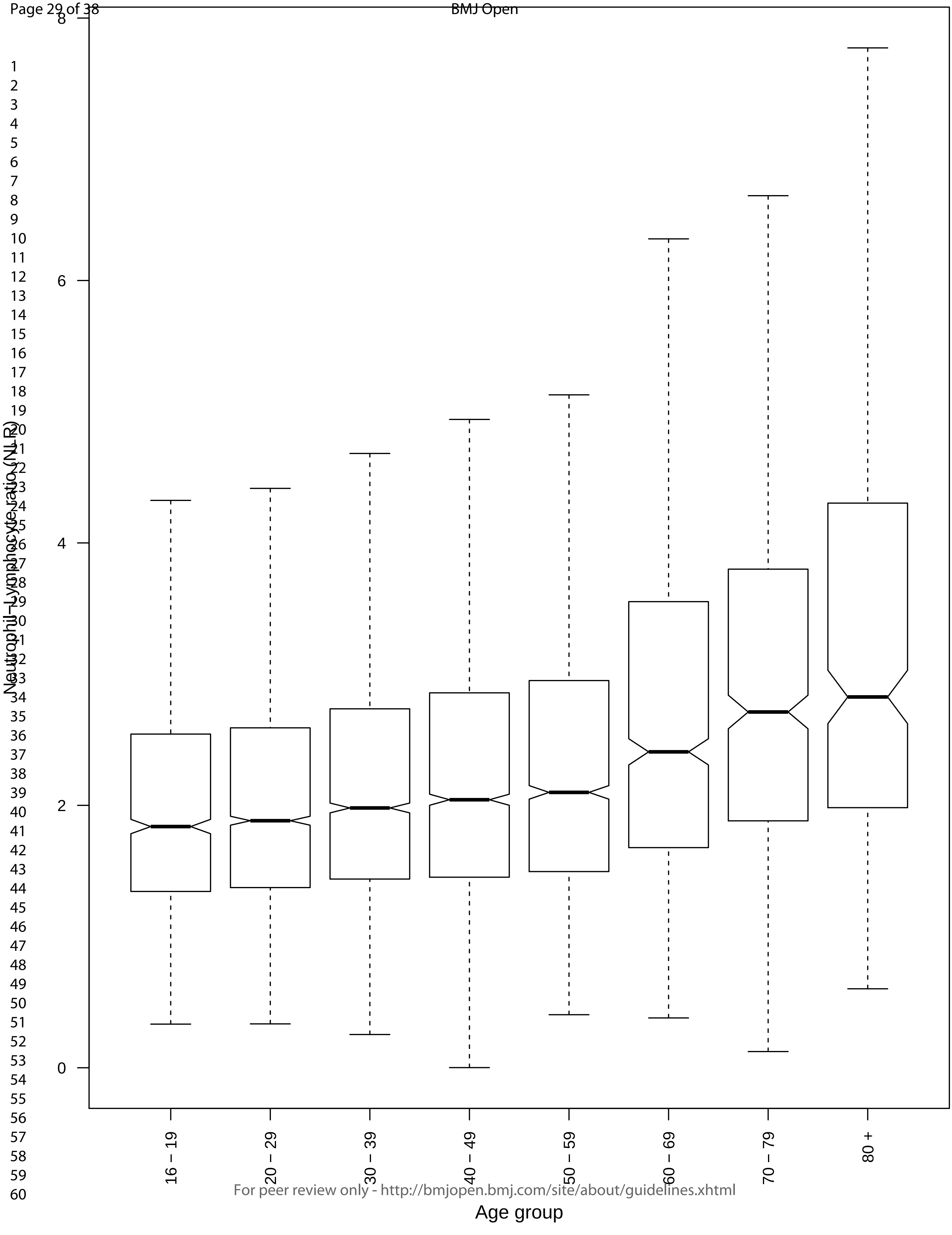
Neutrophil–Lymphocyte ratio (NLR)
Age group
8
6
4
2
0
16 – 19
20 – 29
30 – 39
40 – 49
50 – 59
60 – 69
70 – 79
80 +

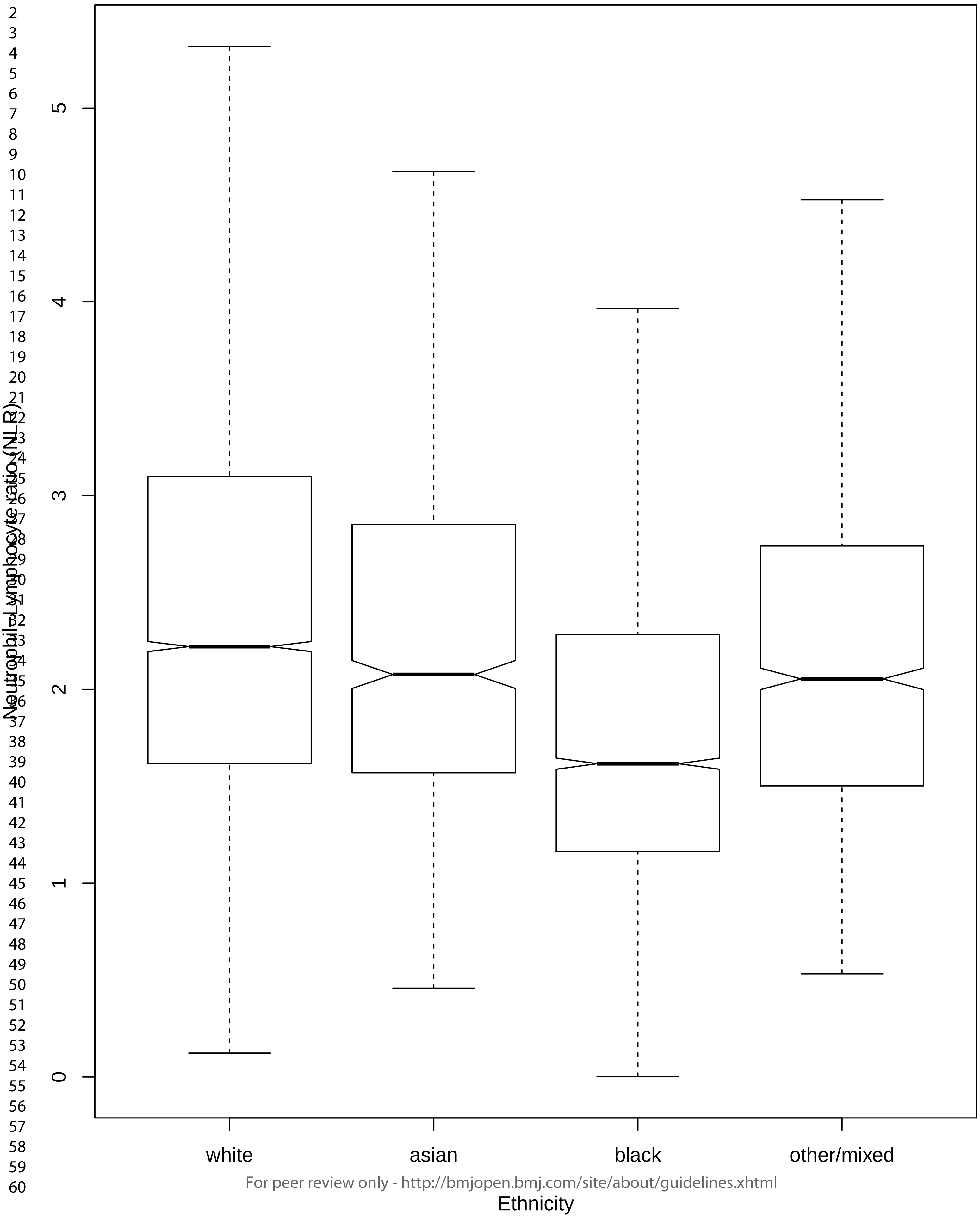
Neutrophil-Lymphocyte ratio (NLR)
5
4
3
2
1
0
white
asian
black
other/mixed
Ethnicity

Supplementary Materials

Table S1: NLR between controls and ICD-10 diagnoses using a log-linked gamma generalised linear model.

	Estimate	Std. Error	z value	Pr(>|z|)	
(Intercept)	0.5243	0.023	22.55	< 2e-16	***
F00	0.1483	0.031	4.834	1.34E-06	***
F01	0.2366	0.081	2.922	0.00348	**
F02	0.2687	0.101	2.657	0.00789	**
F03	0.1166	0.064	1.832	0.06699	.
F04	-0.455	0.283	-1.61	0.10796	
F05	0.2782	0.094	2.964	0.00303	**
F06	0.1643	0.048	3.431	0.0006	***
F07	0.1374	0.101	1.362	0.17319	
F09	0.0506	0.108	0.47	0.63829	
F10	0.158	0.021	7.649	2.03E-14	***
F11	0.0185	0.037	0.504	0.614	
F12	0.043	0.05	0.865	0.38709	
F13	0.0085	0.11	0.077	0.93867	
F14	-0.098	0.091	-1.08	0.28035	
F15	-0.159	0.142	-1.12	0.26347	
F16	-0.039	0.2	-0.2	0.84467	
F17	-0.314	0.49	-0.64	0.52183	
F18	-0.112	0.347	-0.32	0.74688	
F19	0.0642	0.036	1.785	0.07423	.
F20	0.0944	0.018	5.331	9.74E-08	***
F21	-0.168	0.186	-0.9	0.36673	
F22	0.0539	0.041	1.308	0.19077	
F23	0.1595	0.029	5.505	3.69E-08	***
F25	0.0737	0.024	3.067	0.00216	**
F28	0.1657	0.076	2.171	0.02992	*
F29	0.1263	0.024	5.207	1.92E-07	***
F30	0.2002	0.043	4.67	3.01E-06	***
F31	0.1161	0.023	5.123	3.01E-07	***
F32	0.1318	0.02	6.514	7.31E-11	***
F33	0.1439	0.029	4.938	7.89E-07	***
F34	-0.113	0.103	-1.1	0.27259	
F38	0.4065	0.127	3.196	0.00139	**
F39	-0.014	0.116	-0.12	0.90559	
F40	-0.137	0.092	-1.49	0.13529	
F41	0.0666	0.031	2.135	0.03277	*
F42	-0.017	0.033	-0.51	0.60838	
F43	0.0829	0.029	2.825	0.00473	**
F44	0.0348	0.049	0.716	0.47408	

For peer review only

F45	0.0248	0.063	0.397	0.69152	
F48	-0.047	0.035	-1.36	0.17526	
F50	0.0274	0.016	1.747	0.08069	.
F52	-0.206	0.2	-1.03	0.30387	
F53	0.0962	0.056	1.728	0.084	.
F55	-0.093	0.49	-0.19	0.84988	
F60	0.0883	0.025	3.476	0.00051	***
F61	0.1759	0.083	2.127	0.03343	*
F64	-0.105	0.283	-0.37	0.7107	
F68	0.1445	0.347	0.417	0.67677	
F69	0.0032	0.186	0.017	0.98645	
F70	0.1351	0.058	2.344	0.01909	*
F71	0.097	0.113	0.855	0.39248	
F72	0.101	0.245	0.412	0.68036	
F78	0.192	0.49	0.392	0.69525	
F79	0.3979	0.201	1.984	0.04731	*
Age	0.005	2E-04	20.22	< 2e-16	***
Black ethnicity	-0.226	0.019	-11.9	< 2e-16	***
Mixed/other ethnicity	-0.022	0.021	-1.07	0.28411	
White ethnicity	0.0921	0.018	4.998	5.79E-07	***
Gender Male	-0.01	0.008	-1.31	0.19134	
Gender Not Specified	-0.425	0.347	-1.22	0.22082	
Gender Other	-0.096	0.49	-0.2	0.84531	
Antipsychotics	0.0375	0.013	2.963	0.00305	**
Antidepressants	0.0115	0.009	1.247	0.21246	
Mood stabilisers	-0.004	0.016	-0.23	0.81925	
Hypnotics	0.025	0.01	2.54	0.01107	*

Signif. codes: ‘***’ 0.001 ‘**’ 0.01 ‘*’ 0.05 ‘.’ 0.1

Table S2: NLR between controls and ICD-10 diagnoses using a log-linked gamma generalised linear model uncorrected for covariates.

	Estimate	Std. Error	z value	Pr(>|z|)	
(Intercept)	0.73521	0.00818	89.878	< 2e-16	***
F00	0.39918	0.03013	13.25	< 2e-16	***
F01	0.50716	0.08349	6.075	1.24E-09	***
F02	0.45036	0.10486	4.295	1.75E-05	***
F03	0.39547	0.06504	6.08	1.20E-09	***
F04	-0.42323	0.29581	-1.431	0.152494	
F05	0.49682	0.09713	5.115	3.14E-07	***
F06	0.22881	0.04887	4.682	2.84E-06	***
F07	0.26206	0.10486	2.499	0.012453	*

For peer review only

F09	0.07505	0.11206	0.67	0.50304	
F10	0.26071	0.01911	13.64	< 2e-16	***
F11	0.10116	0.03687	2.744	0.006068	**
F12	-0.03256	0.05018	-0.649	0.516471	
F13	0.08988	0.11481	0.783	0.433742	
F14	-0.07706	0.09386	-0.821	0.411629	
F15	-0.08964	0.14807	-0.605	0.544945	
F16	-0.05719	0.20925	-0.273	0.784626	
F17	-0.1782	0.51222	-0.348	0.727919	
F18	-0.20156	0.36224	-0.556	0.577916	
F19	0.08136	0.03628	2.243	0.024911	*
F20	0.09383	0.01256	7.47	8.04E-14	***
F21	-0.10163	0.19375	-0.525	0.599898	
F22	0.14609	0.04094	3.568	0.000359	***
F23	0.09937	0.02676	3.713	0.000205	***
F25	0.06628	0.02038	3.252	0.001144	**
F28	0.14642	0.07853	1.864	0.062257	.
F29	0.06177	0.02095	2.948	0.003196	**
F30	0.19001	0.04234	4.488	7.20E-06	***
F31	0.16235	0.01815	8.944	< 2e-16	***
F32	0.19281	0.01779	10.838	< 2e-16	***
F33	0.26541	0.02785	9.528	< 2e-16	***
F34	-0.05149	0.10711	-0.481	0.630725	
F38	0.55561	0.13249	4.194	2.75E-05	***
F39	0.03868	0.12099	0.32	0.749236	
F40	-0.10782	0.09546	-1.13	0.258666	
F41	0.1576	0.03059	5.153	2.57E-07	***
F42	0.06606	0.03239	2.039	0.041423	*
F43	0.0728	0.02876	2.531	0.011369	*
F44	0.10234	0.04996	2.049	0.040501	*
F45	0.08563	0.06454	1.327	0.184609	
F48	0.01074	0.03595	0.299	0.765057	
F50	0.03339	0.01392	2.399	0.016452	*
F52	-0.17707	0.20925	-0.846	0.397441	
F53	0.09034	0.05648	1.6	0.109689	
F55	0.05383	0.51222	0.105	0.9163	
F60	0.15522	0.02323	6.682	2.35E-11	***
F61	0.20589	0.08575	2.401	0.01635	*
F64	-0.12422	0.29581	-0.42	0.674545	
F68	0.17984	0.36224	0.496	0.619568	
F69	0.02839	0.19375	0.147	0.883506	
F70	0.15574	0.05894	2.642	0.008231	**
F71	0.09172	0.11778	0.779	0.436162	
F72	0.18543	0.25621	0.724	0.469227	
F78	0.27214	0.51222	0.531	0.595216	
F79	0.53105	0.20925	2.538	0.011152	*

For peer review only

Signif. codes: '***' 0.001 '**' 0.01 '*' 0.05 '.' 0.1

Table S3: NLR between ICD-10 diagnoses with elevated NLR in first model using a log-linked gamma generalised linear model.

	Estimate	Std. Error	z value	Pr(>|z|)	
(Intercept)	0.6081	0.0453	13.412	< 2e-16	***
F01	0.0927	0.0872	1.064	0.28742	
F02	0.1411	0.1075	1.312	0.18937	
F05	0.1351	0.1001	1.351	0.17685	
F06	0.0472	0.0567	0.834	0.40448	
F10	0.0371	0.0359	1.033	0.30155	
F20	-0.0104	0.0337	-0.309	0.75758	
F23	0.0608	0.0423	1.44	0.14991	
F25	-0.0333	0.0376	-0.887	0.37531	
F28	0.0617	0.0841	0.734	0.46269	
F29	0.0288	0.0391	0.736	0.46202	
F30	0.0979	0.0532	1.839	0.06587	.
F31	0.0027	0.0366	0.074	0.9411	
F32	0.0115	0.0357	0.323	0.74672	
F33	0.0154	0.0407	0.378	0.7053	
F38	0.2727	0.1344	2.029	0.0425	*
F41	-0.0555	0.0435	-1.278	0.20121	
F43	-0.027	0.0427	-0.632	0.52728	
F60	-0.0299	0.0395	-0.757	0.44903	
F70	0.0292	0.0662	0.441	0.65913	
F79	0.2722	0.2093	1.301	0.19336	
Age	0.0059	0.0004	16.212	< 2e-16	***
Black ethnicity	-0.2412	0.022	-10.945	< 2e-16	***
Mixed/other ethnicity	-0.0005	0.0262	-0.019	0.98449	
White ethnicity	0.1052	0.0215	4.897	9.73E-07	***
Gender Male	-0.0322	0.0105	-3.069	0.00215	**
Gender Not Specified	-0.1816	0.5072	-0.358	0.72033	
Antipsychotics	0.0331	0.0161	2.055	0.03992	*
Antidepressants	0.0134	0.0113	1.187	0.23507	
Mood stabilisers	-0.0079	0.0168	-0.472	0.63677	
Hypnotics	0.0299	0.0113	2.635	0.00842	**

Signif. codes: '***' 0.001 '**' 0.01 '*' 0.05 '.' 0.1

For peer review only

Table S4: NLR between ICD-10 diagnoses with elevated NLR in first model using a log-linked gamma generalised linear model uncorrected for covariates.

	Estimate	Std. Error	z value	Pr(>|z|)	
(Intercept)	1.1344	0.03027	37.471	< 2e-16	***
Short_DiagF01	0.10798	0.09188	1.175	0.239912	
Short_DiagF02	0.05117	0.11327	0.452	0.651437	
Short_DiagF05	0.09764	0.10549	0.926	0.354694	
Short_DiagF06	-0.17037	0.05871	-2.902	0.00371	**
Short_DiagF10	-0.13847	0.03524	-3.929	8.52E-05	***
Short_DiagF20	-0.30535	0.03187	-9.582	< 2e-16	***
Short_DiagF23	-0.29982	0.0403	-7.439	1.01E-13	***
Short_DiagF25	-0.3329	0.036	-9.246	< 2e-16	***
Short_DiagF28	-0.25276	0.08699	-2.906	0.003663	**
Short_DiagF29	-0.33741	0.03636	-9.28	< 2e-16	***
Short_DiagF30	-0.20917	0.05289	-3.955	7.67E-05	***
Short_DiagF31	-0.23684	0.03468	-6.829	8.55E-12	***
Short_DiagF32	-0.20638	0.03448	-5.986	2.15E-09	***
Short_DiagF33	-0.13377	0.0411	-3.255	0.001135	**
Short_DiagF38	0.15643	0.14135	1.107	0.268444	
Short_DiagF41	-0.24158	0.04317	-5.596	2.19E-08	***
Short_DiagF43	-0.32638	0.04178	-7.812	5.61E-15	***
Short_DiagF60	-0.24396	0.03784	-6.448	1.14E-10	***
Short_DiagF70	-0.24345	0.06805	-3.578	0.000347	***
Short_DiagF79	0.13187	0.2204	0.598	0.549634	

Signif. codes: '***' 0.001 '**' 0.01 '*' 0.05 '.' 0.1

Table S5: Association between NLR and mortality using a log-linked gamma generalised linear model.

	Estimate	Std. Error	z value	Pr(>|z|)	
(Intercept)	0.629	0.042	15.05	< 2e-16	***
Mortality	0.103	0.019	5.547	2.90E-08	***
F01	0.075	0.086	0.881	0.378368	
F02	0.112	0.106	1.06	0.289297	
F03	-0.03	0.069	-0.44	0.662089	
F04	-0.55	0.288	-1.92	0.054981	.
F05	0.123	0.098	1.251	0.211037	
F06	0.048	0.055	0.867	0.385872	
F07	0.01	0.106	0.092	0.9267	
F09	-0.06	0.113	-0.5	0.617792	
F10	0.04	0.035	1.139	0.254815	

For peer review only

F11	-0.1	0.046	-2.19	0.028768	*
F12	-0.05	0.058	-0.93	0.352593	
F13	-0.09	0.115	-0.79	0.427049	
F14	-0.2	0.096	-2.06	0.039388	*
F15	-0.26	0.147	-1.75	0.080089	.
F16	-0.14	0.205	-0.68	0.499768	
F17	-0.43	0.498	-0.86	0.391132	
F18	-0.21	0.353	-0.6	0.5495	
F19	-0.04	0.046	-0.84	0.40397	
F20	-0.01	0.032	-0.3	0.760888	
F21	-0.27	0.19	-1.41	0.159867	
F22	-0.06	0.049	-1.27	0.20328	
F23	0.061	0.041	1.495	0.134955	
F25	-0.03	0.036	-0.86	0.392265	
F28	0.061	0.082	0.747	0.45498	
F29	0.027	0.037	0.709	0.478204	
F30	0.098	0.052	1.895	0.058073	.
F31	0.007	0.035	0.197	0.844041	
F32	0.021	0.035	0.608	0.543242	
F33	0.025	0.04	0.623	0.533192	
F34	-0.22	0.108	-2.07	0.038898	*
F38	0.29	0.132	2.199	0.027893	*
F39	-0.12	0.121	-0.98	0.327305	
F40	-0.24	0.098	-2.47	0.013716	*
F41	-0.04	0.042	-1.04	0.300219	
F42	-0.12	0.044	-2.73	0.006259	**
F43	-0.02	0.041	-0.51	0.611605	
F44	-0.07	0.057	-1.31	0.191077	
F45	-0.09	0.07	-1.25	0.211284	
F48	-0.16	0.046	-3.37	0.000762	***
F50	-0.08	0.035	-2.42	0.015461	*
F52	-0.3	0.205	-1.47	0.141943	
F53	-0.01	0.063	-0.22	0.826974	
F55	-0.19	0.498	-0.39	0.697018	
F60	-0.02	0.038	-0.57	0.571013	
F61	0.076	0.088	0.862	0.388455	
F64	-0.21	0.289	-0.72	0.472579	
F68	0.052	0.352	0.149	0.881745	
F69	-0.1	0.19	-0.53	0.598212	
F70	0.036	0.065	0.559	0.576053	
F71	9E-04	0.118	0.008	0.993735	
F72	0.001	0.25	0.005	0.996367	
F78	0.102	0.498	0.205	0.837852	
F79	0.289	0.205	1.411	0.158226	
Age	0.005	3E-04	16.29	< 2e-16	***
Black ethnicity	-0.24	0.02	-12.1	< 2e-16	***

For peer review only

Mixed/other ethnicity	-0	0.023	-0.16	0.87651	
White ethnicity	0.094	0.019	4.975	6.53E-07	***
Gender Male	-0.03	0.009	-3.14	0.00168	**
Gender Not Specified	-0.42	0.352	-1.2	0.229188	
Gender Other	-0.09	0.497	-0.19	0.853287	
Antipsychotics	0.037	0.013	2.887	0.003889	**
Antidepressants	0.008	0.009	0.88	0.378798	
Mood stabilisers	-0.01	0.016	-0.32	0.748271	
Hypnotics	0.025	0.01	2.479	0.013183	*

Signif. codes: '***' 0.001 '**' 0.01 '*' 0.05 '.' 0.1

For peer review only

1. STROBE Statement—Checklist of items that should be included in reports of *cross-sectional studies*

	Item No	Recommendation
**Title and abstract**	1	(*a*) Indicate the study's design with a commonly used term in the title or the abstract
		(*b*) Provide in the abstract an informative and balanced summary of what was done and what was found
**Introduction**		
Background/rationale	2	Explain the scientific background and rationale for the investigation being reported
Objectives	3	State specific objectives, including any prespecified hypotheses
**Methods**		
Study design	4	Present key elements of study design early in the paper
Setting	5	Describe the setting, locations, and relevant dates, including periods of recruitment, exposure, follow-up, and data collection
Participants	6	(*a*) Give the eligibility criteria, and the sources and methods of selection of participants
Variables	7	Clearly define all outcomes, exposures, predictors, potential confounders, and effect modifiers. Give diagnostic criteria, if applicable
Data sources/ measurement	8*	For each variable of interest, give sources of data and details of methods of assessment (measurement). Describe comparability of assessment methods if there is more than one group
Bias	9	Describe any efforts to address potential sources of bias
Study size	10	Explain how the study size was arrived at
Quantitative variables	11	Explain how quantitative variables were handled in the analyses. If applicable, describe which groupings were chosen and why
Statistical methods	12	(*a*) Describe all statistical methods, including those used to control for confounding
		(*b*) Describe any methods used to examine subgroups and interactions
		(*c*) Explain how missing data were addressed
		(*d*) If applicable, describe analytical methods taking account of sampling strategy
		(*e*) Describe any sensitivity analyses
**Results**		
Participants	13*	(a) Report numbers of individuals at each stage of study—eg numbers potentially eligible, examined for eligibility, confirmed eligible, included in the study, completing follow-up, and analysed
		(b) Give reasons for non-participation at each stage
		(c) Consider use of a flow diagram
Descriptive data	14*	(a) Give characteristics of study participants (eg demographic, clinical, social) and information on exposures and potential confounders
		(b) Indicate number of participants with missing data for each variable of interest
Outcome data	15*	Report numbers of outcome events or summary measures
Main results	16	(*a*) Give unadjusted estimates and, if applicable, confounder-adjusted estimates and their precision (eg, 95% confidence interval). Make clear which confounders were adjusted for and why they were included
		(*b*) Report category boundaries when continuous variables were categorized
		(*c*) If relevant, consider translating estimates of relative risk into absolute risk for a meaningful time period

For peer review only

Other analyses	17	Report other analyses done—eg analyses of subgroups and interactions, and sensitivity analyses
**Discussion**		
Key results	18	Summarise key results with reference to study objectives
Limitations	19	Discuss limitations of the study, taking into account sources of potential bias or imprecision. Discuss both direction and magnitude of any potential bias
Interpretation	20	Give a cautious overall interpretation of results considering objectives, limitations, multiplicity of analyses, results from similar studies, and other relevant evidence
Generalisability	21	Discuss the generalisability (external validity) of the study results
**Other information**		
Funding	22	Give the source of funding and the role of the funders for the present study and, if applicable, for the original study on which the present article is based

*Give information separately for exposed and unexposed groups.

Note: An Explanation and Elaboration article discusses each checklist item and gives methodological background and published examples of transparent reporting. The STROBE checklist is best used in conjunction with this article (freely available on the Web sites of PLoS Medicine at http://www.plosmedicine.org/, Annals of Internal Medicine at http://www.annals.org/, and Epidemiology at http://www.epidem.com/). Information on the STROBE Initiative is available at www.strobe-statement.org.

For peer review only

For peer review only - http://bmjopen.bmj.com/site/about/guidelines.xhtml
